# Integrative computational analysis of plant-derived flavonoids as inhibitors of Listeriolysin O and Internalin A in *Listeria monocytogenes*

**DOI:** 10.1371/journal.pone.0351129

**Published:** 2026-06-09

**Authors:** Shahadul Hassan Sourav, Noimul Hasan Siddiquee, Rahnuma Tabassum, Lamia Islam, Syed Kashif Zaidi, Mohammad Sharif Uddin, Sumita Rani Saha, Imam Hossain

**Affiliations:** 1 Department of Microbiology, Noakhali Science and Technology University, Noakhali, Bangladesh; 2 Bioinformatics Laboratory (BioLab), Noakhali, Bangladesh; 3 Department of Biochemistry and Molecular Biology, Jagannath University, Dhaka, Bangladesh; 4 Institute of Genomic Medicine Sciences, King Abdulaziz University, Jeddah, Saudi Arabia‌‌; Kwara State University, NIGERIA

## Abstract

Foodborne pathogen *Listeria monocytogenes* causes listeriosis, a rare but deadly condition. Internalin A (*InlA*) and Listeriolysin O (*LLO*), its main virulence factors, facilitate adhesion, invasion, intracellular survival, and intercellular spreading, making them interesting therapeutic targets. *L. monocytogenes* infections are becoming harder to treat because of antibiotic resistance; hence, flavonoids are being considered. An integrated *in-silico* technique was used to test plant-derived flavonoids’ inhibitory efficacy against these proteins. For both targets, three modes of docking (HTVS, SP, and XP docking) were used for the preliminary screening from a library of 1,254 flavonoids. While CIDs 441667, 15126294, and 187808 showed favorable *in-silico* profiles for *InlA* with scores of −8.461, −7.578, and −7.521 kcal/mol, respectively, CIDs 441699, 443648, and 442868 showed the best affinity for *LLO* with values of −7.446, −5.991, and −5.852 kcal/mol, respectively. Admet analysis predicted the drug-likeness and safety characteristics of the compounds. Subsequently, the QM calculation was employed to examine the interaction of these compounds with the receptor, alongside their MEP and NBO characteristics. The selected ligands and the control ampicillin for both proteins were utilized to build protein-ligand complexes, subsequently assessed via a 100 ns molecular dynamics simulation. Subsequent post-simulation MM-GBSA, PCA and DCCM analysis of the trajectories evaluated their dynamic stability concerning *InlA* and *LLO*. CIDs 441667 and 15126294 for *InlA*, as well as CIDs 441699 and 443648 for *LLO*, have been identified as potential inhibitors, establishing a basis for future in vivo investigations and experimental validation.

## 1. Introduction

Foodborne pathogens cause significant illness by contaminating food throughout the supply chain, including *Salmonella*, *Campylobacter*, *E. coli*, *Listeria*, *S. aureus, Klebsiella*, and *Pseudomonas* [1–4]. Among the 21 identified *Listeria* species [5], *L. monocytogenes* is a ubiquitous bacterium responsible for listeriosis in humans, animals, and poultry, representing a significant public health risk [6–9]. *L. monocytogenes* causes fatal central nervous system (CNS), septicemic, and maternal-fetal infections, unlike other non-pathogenic *Listeria* species [10]. *L. monocytogenes* was acknowledged as a human pathogen in the 1970s and as a foodborne pathogen in the 1980s [11,12]; since then, 2383 global cases with 487 deaths (around 20% fatality, 1996–2018) [13] and over 1500 U.S. cases with 200 deaths (around 13% fatality, 1998–2023), have been reported [14].

*L. monocytogenes*, a widespread small, gram-positive, facultatively anaerobic, nonsporulating pathogen that demonstrates robust growth on blood agar [15], causing up to 30% mortality in immunocompromised individuals through consumption of contaminated ready-to-eat foods and water [6,16–20]. It can survive and grow under high salinity, low pH, and refrigeration conditions [21,22]. E-cadherin, a vital adhesion molecule at the intestinal barrier, blood-brain barrier, and placenta, operates as the cellular receptor for *InlA*, mediating homophilic cell interactions and binding to the actin cytoskeleton via its cytoplasmic domain [23,24]. The invasiveness of the pathogen is driven by *ActA*-mediated actin-based cell-to-cell dispersion and *InlA*, an LPXTG protein that facilitates epithelial cell entry through its LRRs interacting with E-cadherin [8,24–27].

*L. monocytogenes* may infiltrate both phagocytic and non-phagocytic cells, with its capacity to penetrate non-phagocytic cells being a critical aspect of infection [11]. Upon entry, it evades double-membrane compartments to replicate and disseminate while escaping extracellular defenses, with proper clearance dependent on strong cell-mediated immunity, including TNF-α, IFN-γ, M1 macrophages, MHC, and cytosolic innate responses [8,11,28]. After internalisation, the bacterium uses listeriolysin O *(LLO)* and phospholipases *PlcA* and *PlcB* to rupture the vacuole, enabling escape and driving pathogenesis [11]. *LLO*, a pore-forming toxin encoded by the *hly* gene, facilitates vacuolar escape, induces Ca² ⁺ influx, suppresses macrophage oxidative burst, alters host gene transcription and SUMOylation, and modulates adaptive immunity [8,29–31].

The pathogen causes illnesses ranging from non-invasive mild gastrointestinal symptoms to severe invasive diseases, relying upon host immunity. Invasive infections include pregnancy-associated and neonatal listeriosis, leading to 14% of cases [11], predominantly due to vertical transmission and immunosuppression resulting from progesterone-induced attenuation of T-cell-mediated immunity [32–34]; septicemia (52%); and CNS infections, including meningitis or meningoencephalitis, represent 31% [35,36]. It can invade the CNS via multiple routes: direct penetration of the blood–brain barrier, retrograde movement along nerve axons, or the “Trojan horse” mechanism, where infected phagocytes carry bacteria into the brain [37–40]. Potential entry routes encompass the choroid plexus, ventricles, and brain microvasculature [40,41], resulting in meningitis that mainly affects individuals over 50, usually manifesting with fever, headache, and altered sensorium [40,42]. An *InlA*-dependent mechanism of cranial nerve invasion contributes to the process of meningitis infection [43]. Invasive disease has an incubation period of 11–70 days (an average of 31) [40], and immune-related disorders, such as rheumatoid arthritis (RA) and inflammatory bowel disease (IBD), are also associated with *L. monocytogenes*, potentially due to the bacterium’s ability to manipulate host immune responses [44,45].

Medicinal plants contain various phytochemicals, such as alkaloids, flavonoids, terpenoids, and tannins, with antioxidant and antimicrobial properties, and numerous studies over the past decade have explored their antibacterial potential [46,47]. Secondary plant metabolites, which are necessary for plant structure and metabolism but not growth or reproduction, have shown therapeutic potential for several devastating illnesses [48,49]. Flavonoids, a large family of secondary metabolites present in plants, suppress many damaging illnesses, and some molecules can boost antimicrobial activity [50,51].

Secondary metabolites like flavonoids are crucial for a plant’s defense, appearance, and adaptation, but not for its growth or reproduction [52,53]. They are natural plant pigments with diverse bioactivities that compromise bacterial membranes, energy metabolism, and nucleic acid synthesis, making them effective against many bacteria, especially Gram-positive species like *L. monocytogenes*. Plant flavonoids have antibacterial, antioxidant, antiviral, anticancer, anti-aging, anti-radiation, radical scavenging, anti-inflammatory, and anti-tumor characteristics, according to several studies [54–57]. Flavonoids reduce aging and cellular degradation and protect the brain [58]. Most flavonoids are metabolized in the liver and gastrointestinal tract after intake, then enter the bloodstream and cross the BBB to reach the CNS [59]. Their presence strengthens antibacterial activity, especially when combined with terpenes and phenolic acids [60]. Flavonoids inhibit *L. monocytogenes* by increasing membrane permeability [61], leaking cellular contents, altering energy metabolism-critical ATPase and membrane protein functions [62], and hampering biofilm development and defense [63].

A diverse array of 1,256 flavonoid compounds was extracted from the PubChem database (https://pubchem.ncbi.nlm.nih.gov) for *in-silico* drug development targeting *L. monocytogenes*. The control ligand in this study was ampicillin, a well-known antibiotic used to treat listeriosis, particularly severe infections such *as L. monocytogenes*-induced meningitis [64]. The broad-spectrum beta-lactam aminopenicillin, ampicillin, is widely used to treat susceptible Gram-negative and Gram-positive bacteria [65], including urinary tract infections, skin, respiratory, and soft tissue infections, meningitis often caused by *L. monocytogenes*, intra-abdominal infections, and bacterial endocarditis [66].

Bioinformatics methods, coupled with pharmacokinetic and toxicological evaluations, were applied to investigate the interactions and binding affinity between Ampicillin and flavonoid compounds. This facilitated virtual screening and optimization of future therapeutic prospects through computational approaches utilized to assess natural flavonoids [67]. Furthermore, advanced techniques such as Molecular Dynamics Simulation (MD simulation), post-simulation analyses such as Dynamic Cross-Correlation Matrix (DCCM), and Principal Component Analysis (PCA) provided insights into their dynamic stability and inhibitory potential. Collectively, these findings highlight promising candidates against Internalin A and Listeriolysin O of L. monocytogenes, offering prospects for targeted listeriosis therapy.

## 2. Methods and materials

**Fig 1:**
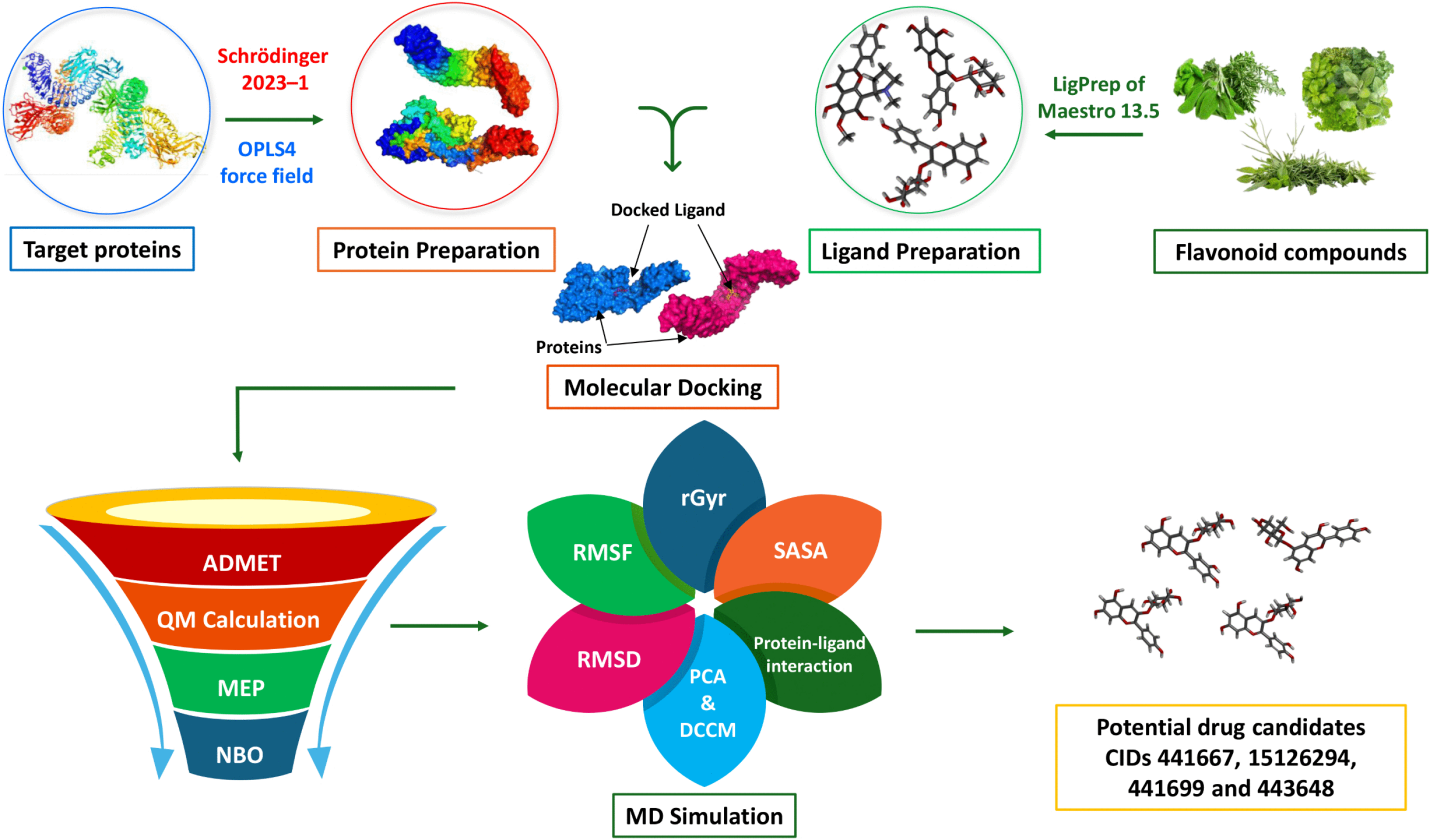
The following figure outlines the overall framework of this study, which aims to investigate L. monocytogenes inhibitors derived from natural flavonoid compounds‌‌.

The overall experimental design and computational workflow used in this study are summarized in Fig 1.

### 2.1. Identification, retrieval, and refining of the target protein

This study employed two target proteins: Internalin A (PDB ID: 8H64) and Listeriolysin O (PDB ID: 4CDB) & the crystal forms of these proteins were obtained from the Protein Data Bank (https://www.rcsb.org/). Internalin A complexed with nanobody VHH24 bound consists of four chains (chain A, C, E, G), 462 amino acids in sequence length, with a resolution of 2.35 Å, a value-free score of 0.227 [68], and Listeriolysin O consists of one chain (chain A), 488 amino acids sequence length with a precision of 2.15 Å, value-free score of 0.246 [69]. Three chains (C, E, G), heteroatoms, and water molecules of Internalin A, as well as the ligands, heteroatoms, and water molecules of Listeriolysin O, were taken out using the Protein Preparation Wizard in Maestro 2023–1. The bond ordering of these proteins was established, and the standard protein preparation parameter, employing the OPLS4 force field, was applied to incorporate missing side chains and hydrogens [70].

### 2.2. Retrieval and optimization of compounds

The anti-listeria antibiotic Ampicillin has been used as a control ligand for the target proteins. The 3D conformer of Ampicillin (CID 6249) was retrieved in SDF format via PubChem (https://pubchem.ncbi.nlm.nih.gov/) and employed as a control. This work utilized 1254 flavonoid compounds in the search for putative inhibitors. The flavonoid compounds were obtained in SDF format through the PubChem website (https://pubchem.ncbi.nlm.nih.gov/) and thereafter processed through the LigPrep function of Maestro v13.5 [71]. The protein and ligands were ultimately tuned using the OPLS4 force field [70]. Additionally, the Epik was employed for reducing the high-energy ionisation states of the ligand molecules to pH 7.0.

### 2.3. Molecular docking: Protein-ligand binding analysis

In structure-based drug design, accurate protein-ligand binding modeling helps uncover new lead compounds and therapeutic candidates. Computational molecular docking methods have successfully adopted the “Lock and Key” idea of protein-ligand binding, which has dominated explanations of these interactions [72]. It is a computer technique based on structural analysis that predicts the binding mechanism and affinity between ligands and targets by simulating their interactions [73]. Docking was conducted between target macromolecules and a unique set of 1,254 flavonoid compounds, along with the control ligand, using the Glide available in Schrödinger Desmond program (Maestro v13.5) in High-Throughput Virtual Screening (HTVS), standard precision (SP), and extra-precision (XP) modes with the OPLS4 force field [70,71]. These modes facilitate the assessment of interactions through various scoring methods, aiming to identify ligands that exhibit the best affinity to the target protein. The native inhibitor was assessed in association with targeted proteins, and the binding location was chosen for receptor grid generation. A grid box of 46 × 46 × 46 Å was centred at (X = −6.012, Y = 8.208, Z = −26.474) for 4CDB, and at (X = 33.223, Y = −23.645, Z = −20.608) for 8H64, based on the key binding site residues of each receptor. The Maestro viewer was used to visualize various forms of chemical bonds and residues interacting with ligands, which were obtained by binding with the energy of target proteins [74]. In this study, two molecular docking runs were performed for each mode (HTVS, SP, and XP) for each target protein. Additionally, the docking protocol was benchmarked using the experimentally validated antibiotic ampicillin as a reference ligand. The binding affinities of screened compounds were compared against this control to evaluate the relative performance and reliability of the docking-based ranking.

### 2.4. Pharmacokinetics (PK) prediction

Computational drug discovery requires pharmacokinetic prediction to understand preclinical failures and how drugs are distributed in the body [75]. The prospective pharmacological and therapeutic efficacy of possible drug candidates is primarily influenced by absorption, distribution, metabolism, and excretion (adme). The pharmacological characteristics were assessed by the top docking-scored ligand molecules, along with the control, via the pkCSM tool (https://biosig.lab.uq.edu.au/pkcsm/) [76]. Lipinski’s rule of five and gastrointestinal (GI) permeability are all critical factors in rational drug design, and this system evaluates the compound’s ability to meet these parameters [75].

### 2.5. Toxicity profiles prediction

Small molecules can also be toxic to multiple organs in the human body, so various toxicological parameters, including anticipated LD50, predicted toxicity class, and other endpoints, as well as potential organ toxicity, must be considered. Thus, the primary focus of drug development is toxicity prediction. ProTox (https://tox.charite.de/protox3/) 3.0 was employed in this investigation to forecast the toxicity and, in addition, the penetration of the top docking-scored compounds through the blood-brain barrier (BBB) [77].

### 2.6. Quantum mechanical (QM) calculation

Density functional theory (DFT) represents a quantum mechanical (QM) approach to determining the electronic structure of solids, molecules, and atoms [78]. In the ligand QM calculation, DFT was applied to isolated ligands for electronic structure analysis [79]. The Gaussian 09W and GaussView 6.0 software were used as computational tools for optimizing the geometry of molecular structures using density functional three-parameter hybrid (B3LYP) approaches [80]. In the DFT study, frontier molecular orbitals were observed, comprising the highest occupied molecular orbitals (HOMOs) and the lowest unoccupied molecular orbitals (LUMOs), along with the energy gap between them. The energy values of HOMOs indicate a ligand molecule’s potential for donating electrons, whereas the energy levels of LUMOs suggest the molecule’s its capability to accept from the protein [81]. The HOMO-LUMO gap (HLG) serves as an effective indicator of a molecule’s electrophilic index, chemical potential, and its characteristics of softness or hardness [82]. The following equation was used to compute the hardness, softness, and gap energies from the frontier energies (ε) of HOMOs and LUMOs.

Hardness(η) = (I − A)/2
Softness(S) = 1/η

Here, “I” stands for the ionization potential (− E_HOMO_) & the electron affinity (− E_LUMO_) represented by “A” in the equation. The preceding equation indicates that a lower hardness value equates with increased reactivity, and conversely. Soft molecules with low HOMO-LUMO gap energy are chemically reactive but kinetically unstable. A molecule with a high HOMO-LUMO gap must have limited chemical or bioactivity and excellent kinetic stability because the high-energy LUMO rarely acquires an electron. Furthermore, B3LYP methods along with the 6-311G basis set were used to assess the molecular electrostatic potential (MEP) and natural bond orbital (NBO) analysis.

### 2.7. Evaluation of molecular dynamics (MD) simulation

MD simulation are essential for a thorough comprehension of the activity and integrity of protein-ligand complexes in diverse biological contexts [74]. These simulation are essential for evaluating the binding stability and activity within the active site cavity by providing a dynamic understanding of the interactions among the ligands and protein [81]. Our investigation involved molecular dynamics simulation extending 100 nanoseconds, utilizing the Desmond module from Schrödinger (Release 2023−1) within a Linux environment [83]. The system was solvated in an orthorhombic periodic boundary box with a 10 Å buffer distance using the simple point-charge (SPC) water model, while maintaining a 0.15 M salt concentration with randomly distributed Na⁺ and Cl⁻ ions. The total system size consisted of approximately 48,100 atoms for the 4CDB complex and 44,500 atoms for the 8H64 complex, respectively. Convergence was monitored through system parameters. The system was minimized and equilibrated using the OPLS4 force field [70,84]. Lastly, the constant pressure-constant temperature (NPT) ensemble was performed at 300 K and 1.01325 bar using a Nose-Hoover chain thermostat (relaxation time: 1.0 ps) and a Martyna-Tobias-Klein barostat with isotropic coupling (relaxation time: 2.0 ps) [81]. Following the system’s relaxation for every complex, the final cycle of production was executed with recording intervals of 100 ps and an energy level of 1.2.

## 3. Result

### 3.1. Study of molecular docking

**Fig 2:**
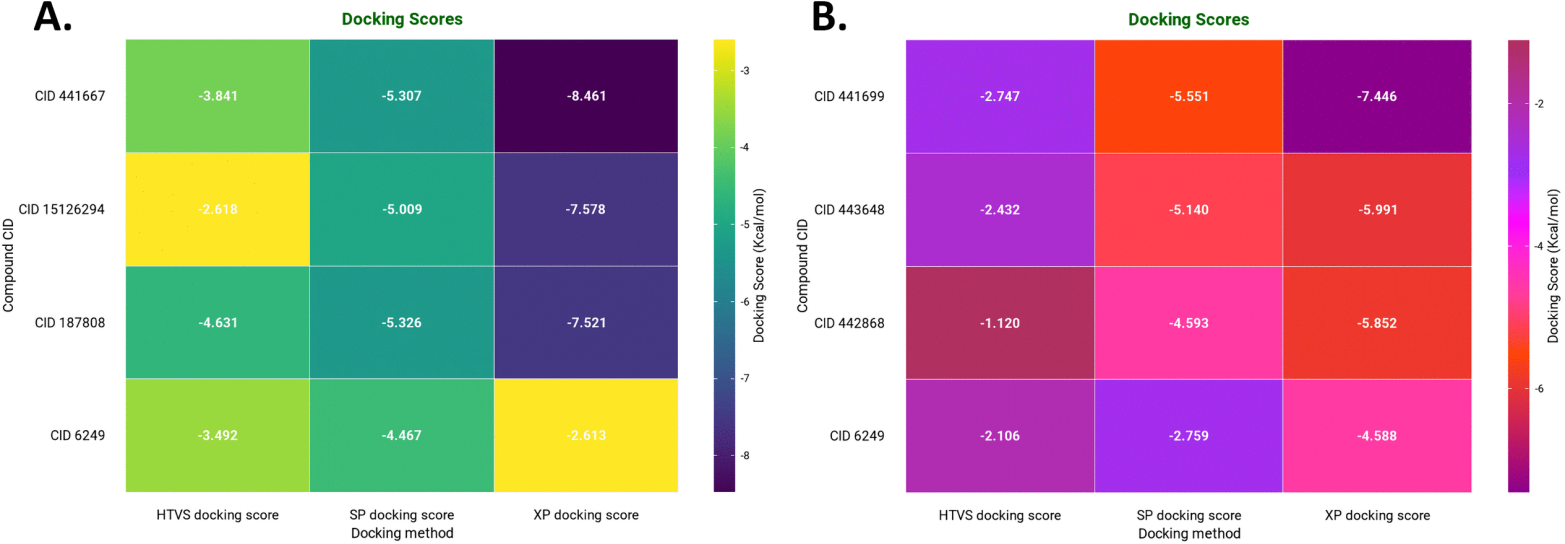
Molecular docking scores (kcal/mol) of the six hit compounds and native ligand against 4CDB **(A)** and 8H64 proteins **(B)** were inspected using different docking approaches. Here, the first column implies the high-throughput virtual screening (HTVS) scores, the second column corresponds to the standard precision (SP) scores, and the third column represents the extra precision (XP) docking scores for the similar compounds.

HTVS, SP docking, and XP docking were among the molecular docking techniques employed in this study to assess the binding interactions of small-molecule drugs with the target proteins. After XP docking, out of 1254 compounds, three compounds had better docking scores than Ampicillin (control) for each of the two proteins. Against the 4CDB protein, three compounds were selected, including CIDs 441667, 15126294, and 187808, which require further investigation as they have a higher tendency for binding with docking scores −8.461, −7.578, and −7.521 kcal/mol, respectively, than the control CID 6249 (−2.613) (Table 1) (Fig 2). Against the 8H64 protein, three compounds were selected, including CIDs 441699, 443648, and 442868, for additional evaluation as they have an elevated binding affinity with docking scores −7.446, −5.991, and −5.852 kcal/mol, respectively, than the control CID 6249 (−4.588) (Table 1) (Fig 2). Based on the docking scores of the control as a cut-off, the top three compounds were selected as leads for each target, prioritizing safety, pharmacokinetics, and docking scores. The identified compounds demonstrated significantly better docking scores compared to the reference drug ampicillin, further supporting the reliability of the docking-based ranking.

**Table 1:** The biological uses for the six selected flavonoids, as well as Ampicillin (control), against the two target proteins (4CDB and 8H64), including the compound’s name, identification, and origin (plant).

Protein 4CDB			
PubChem CID	Compound Name	Plant Source	Biological Use
441667	Cyanidin 3-glucoside	Blackberries [85], Red Grape [86], Concord Grape [86], *Allium Cepa* [87].	Antioxidant [88], anti-inflammatory [89], anti-obesity [90], Anticarcinogenic [91].
15126294	Cyanidin 5-O-glucoside	N/A	N/A
187808	Glycitein 7-O-glucoside	*Glycine max* [92]	Antioxidants [92]
6249 (Control)	Ampicillin	N/A	Anti-bacterial [93] Anti-gynecological infections [94]
**Protein 8H64**			
**PubChem CID**	**Compound Nam**	**Plant Sourc**	**Biological Use**
441699	Cyanidin 3-O-galactoside	*Crataegus spp.* [95], *Aronia* plants [96], *Arbutus unedo* [97], Purple wheat [98], Black soybean seed coat (*Glycine max L. Merr.*) [99].	Anti-Oxidant [100], Anti-cancer [101], Anti-inflammatory [102], Anti-diabetic [103], Cardiovascular protective [104].
443648	Pelargonidin 3-glucoside ion	Strawberry [105]	Neuroprotective [106], Antioxidant [107], anti-inflammatory [108], anti-obesity [109], anti-cancer [110], antidiabetic [111].
442868	Phyllospadine	*Phyllosphadix iwatensis* [112]	N/A
6249 (Control)	Ampicillin	N/A	Anti-bacterial [93], Anti-gynecological infections [94].

### 3.2. Evaluation of pharmacokinetic (PK) properties

**Fig 3:**
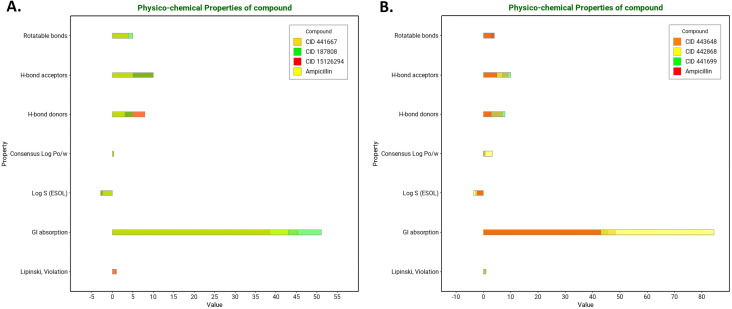
Summary of pharmacokinetic characteristics for chosen drugs. Herein, **(A)** presents an in-depth assessment of the pharmacokinetic properties of the three chosen compounds against the 4CDB protein, whereas (B) presents a detailed analysis of the pharmacokinetic features of the three selected compounds against the 8H64 protein, along with the control drug (Ampicillin). It involves a wide variety of parameters, including pharmacokinetics, physicochemical qualities, water solubility, lipophilicity, and drug-likeness.

The pharmacokinetic properties of the chosen lead compounds for 4CDB protein (CIDs 441667, 15126294, 187808) and 8H64 protein (CIDs 441699, 443648, 442868), as well as the control (CID 6249), were assessed using Lipinski’s rule of five, presented in Fig 3 and S1 File, respectively. All selected lead compounds complied with all five of Lipinski’s criteria, indicating they all possessed potential drug-like properties. Their high permeability of the gastrointestinal tract indicates higher bioavailability at the target site. These features suggest positive pharmacokinetic profiles that may reduce the risk of clinical trial failure.

### 3.3. Evaluation of potential toxic effects

**Fig 4:**
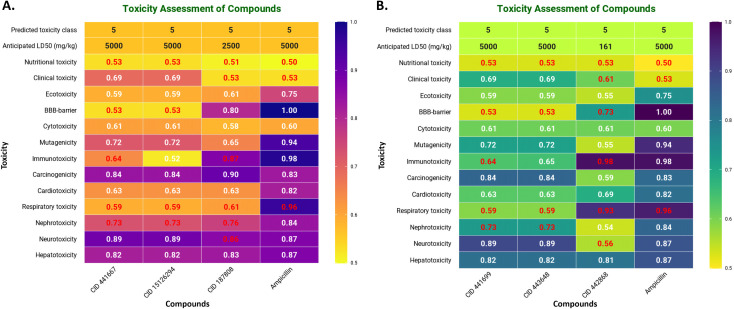
Overview of toxicity properties for selected compounds. Here, **(A)** offers a thorough evaluation of the diverse properties of the three selected compounds against the 4CDB protein, whereas **(B)** provides a detailed analysis of the toxicity features of the three selected compounds against the 8H64 protein, including parameters like Anticipated LD_50_, Predicted toxicity class, toxicity end points, along with blood-brain barrier (BBB) permeability and organ toxicity. Here, the red text shows the active toxicity status of the respective compounds.

Predicting toxicology is a critical component of drug development regulation, as it provides information on the prospective detrimental effects of substances on the environment, animals, and humans. A summary of the results for each drug (CIDs 441667, 15126294, 187808, 441699, 443648, 442868, and 6249) for both proteins (4CDB & 8H64) were illustrated in Fig 4 and listed in the S2 File. These findings indicate that the leads are predicted to have low toxicity based on computational models, providing preliminary *in silico* safety profiles.

### 3.4. Protein-ligand binding interaction

**Fig 5:**
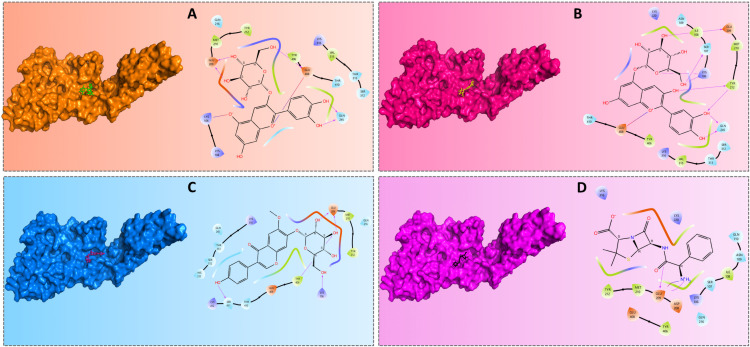
Molecular docking interactions between the Internalin A (4CDB) and the three targeted compounds are represented in both 3D and 2D formats. (**A-D**) interactions of CIDs 441667, 15126294, 187808, and 6249 (control), accordingly.

**Fig 6:**
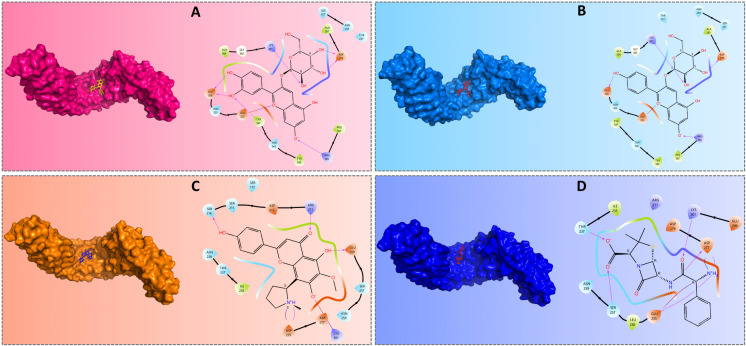
Molecular docking interactions between the Listeriolysin O (8H64) and the three chosen compounds are illustrated in both 3D and 2D formats. (**A-D**) interactions of CIDs 441699, 443648, 442868, and 6249 (control), accordingly.

**Fig 7:**
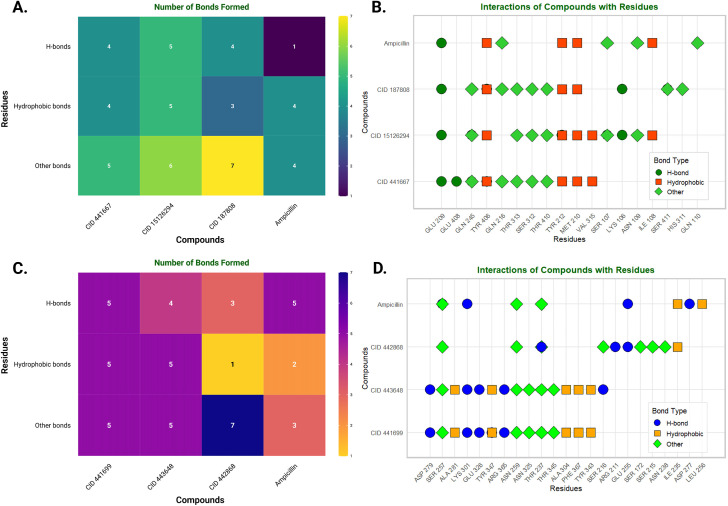
Analysis of residual interactions and closeness of the chosen six compounds with 4CDB & 8H64 protein. Herein, **(A)** and **(C)** show different types and numbers of residual interactions between the six compounds and ampicillin, the control compound. Each bar displays the number of unique interactions and how the taken compounds bind compared to ampicillin. Where **(B)** and **(D)** represent the types of interactions established, the residues particular to proteins and the chosen four chemicals are shown. The proximity of these compounds to proteins helps to pinpoint the specific residues engaged in their interactions.

**Fig 8:**
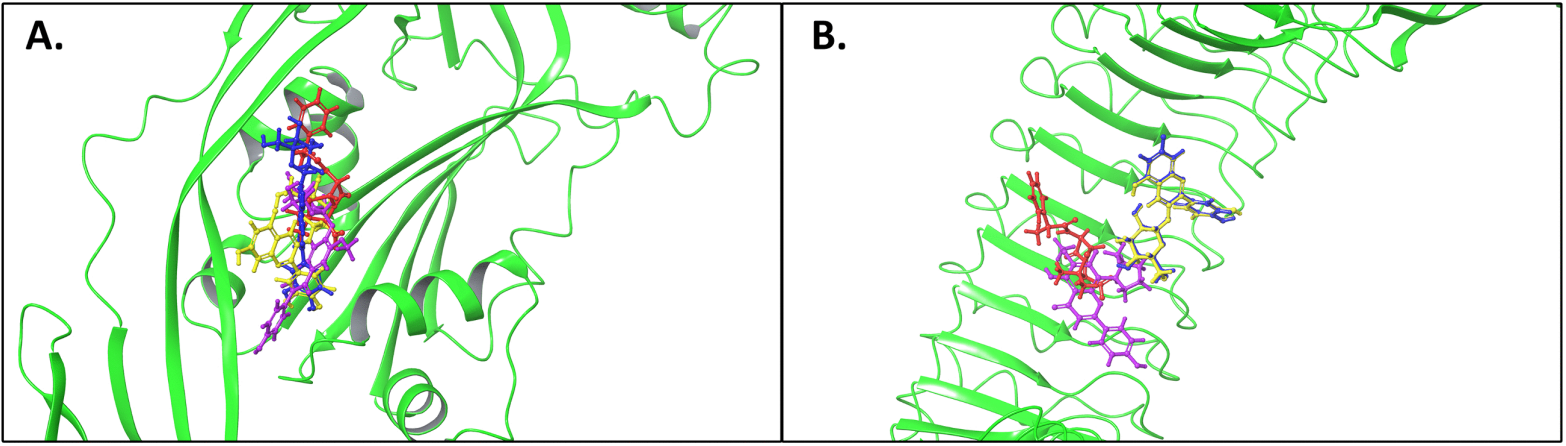
Docking poses of selected flavonoids and control inhibitors within the active sites of **(A)** InA and **(B)** LLO. Control compounds were shown in red; CIDs 441667/441699 in yellow; CIDs 15126294/443648 in blue; and CIDs 187808/442868 in violet. Protein structures were displayed as green ribbon models.

Binding studies were performed utilizing Maestro v13.5 to illustrate the interaction bonds with residues, as depicted in Figs 5–8. The findings of an analysis of the ligand-target protein interactions are shown in Fig 7 and the S3 File. These interactions disclosed a varied spectrum of non-covalent interactions, encompassing hydrophilic, hydrogen bonds, hydrophobic contacts, polar bonds, and electrostatic interactions. The protein-ligand complex’s stability was substantially enhanced by the existence of hydrogen bonds in the interactions.

In the case of the 4CDB protein, CID 15126294 exhibited five hydrogen bonds, which was the highest among the other two compounds, CID 441667 and CID 187808, both of which revealed four hydrogen bonds, whereas the control ligand, ampicillin, exhibited only one hydrogen bond (Fig 7A). It was remarkable that the three ligands exhibited a greater number of hydrogen bonds than the control drugs. All selected chemicals notably interacted with a shared residue, GLU 209, through hydrogen bonding. Furthermore, various other residues, such as TYR 212, TYR 406, and MET 210, were found as commonly interacting residues (Fig 7B). These residues contribute to diverse bonding types, including hydrophobic, ionic, and polar bonds.

Regarding the 8H64 protein, CID 441699 and control ligand ampicillin both exhibited five hydrogen bonds, while CID 443648 and CID 442868 revealed four and three hydrogen bonds, respectively (**Fig 7C**). A similar binding pattern was indicated by the interaction between the control ligand and the top three compounds with multiple residues of amino acids (SER 257, ASN 259, THR 237), presented in **Fig 7D**. These residues contribute to diverse bonding types, including ionic and polar bonds.

### 3.5. Quantum mechanical (QM) calculation

**Fig 9:**
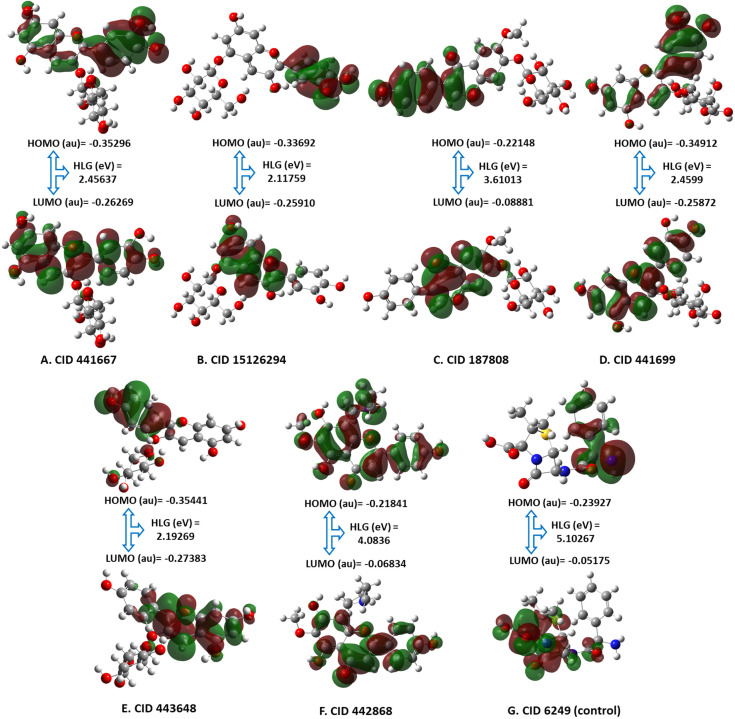
The energy score of the HOMOs and LUMOs of the six lead chemicals and the control drug, against the target 4CDB & 8H64 proteins, including their 3D structures, as well.

Regarding the 4CDB protein, the three principal compounds, CID 441667, CID 15126294, and CID 187808, along with the control (CID 6249), displayed HOMO and LUMO energy scores of (−0.35296 and −0.26269), (−0.33692 and −0.25910), (−0.22148 and −0.08881), and (−0.23927 and −0.05175), respectively, measured in atomic units (a.u.), as illustrated in **Fig 9**. The HLG, hardness, and softness energy scores were determined for the chosen three compounds along with the control, as shown in **Table 2**.

**Table 2:** The HOMO-LUMO analysis was conducted on six selected compounds in comparison to the control drug against the target proteins 4CDB and 8H64.

Compounds CID	HOMO (a.u.)	LUMO (a.u.)	HLG (eV)	Hardness (eV)	Softness (eV^-1^)
441667	−0.35296	−0.26269	2.45637	1.22819	0.81421
15126294	−0.33692	−0.25910	2.11759	1.058795	0.94447
187808	−0.22148	−0.08881	3.61013	1.80506	0.55399
441699	−0.34912	−0.25872	2.4599	1.22995	0.83104
443648	−0.35441	−0.27383	2.19269	1.09635	0.91212
442868	−0.21841	−0.06834	4.0836	2.0418	0.48976
6249	−0.23927	−0.05175	5.10267	2.55133	0.39195

Regarding the 8H64 protein, the three principal compounds, CID 441699, CID 443648, CID 442868 along with the control (CID 6249), exhibited HOMO and LUMO energy scores of (−0.34912 and −0.25872), (−0.35441 and −0.27383), (−0.21841 and −0.06834), and (−0.23927 and −0.05175), accordingly, measured in atomic units (a.u.), as illustrated in **Fig 9**. The HLG, hardness, and softness energy scores were determined for the chosen three compounds along with the control, as shown in **Table 2**.

### 3.6. Molecular electrostatic potential analysis

**Fig 10:**
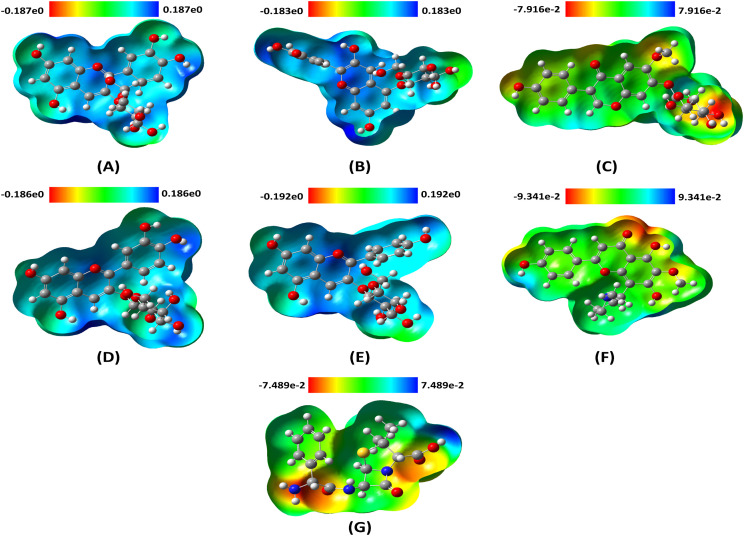
Molecular electrostatic potential (MEP) map of the chosen six compounds CIDs: **(A)** 441667, **(B)** 15126294, **(C)** 187808, **(D)** 441699, **(E)** 443648, and **(F)** 442868, along with control compound **(G)** 6249.

Molecular electrostatic potential (MEP) map facilitates the prediction of a compound’s responsiveness, binding areas, and sites by providing a visual portrayal of the charge dispersion throughout the compound [113]. In computational chemistry, it becomes crucial to identify the areas that are active for nucleophilic and electrophilic attack across all optimized structures [114,115]. This illustrates the electron-deficient region (green), negatively charged electron-rich area (red), and the positively charged portion (blue), highlighting potential sites for ionic interactions, hydrogen bonding, and other noncovalent attractions. The MEP map of the six chosen compounds was displayed in Fig 10. The range of positive electrostatic potential varied from +9.312e-2 to +0.192 a.u., whereas the negative electrostatic potential spanned from −9.312e-2 to −0.192 a.u. (Fig 10).

Among the three selected compounds (CIDs 441667, 15126294, and 187808) regarding 4CDB protein, CID 15126294 (Fig 10B) showed positive potential at +0.183 a.u. and negative potential at −0.183 a.u., the balanced potential range of CID 441667 (Fig 10A) was (+0.187 to −0.187 a.u.). In contrast, CID 187808 (Fig 10C) exhibited the lowest values, spanning from +0.07916 to −0.07916 a.u.

Among the three selected compounds (CIDs 441699, 443648, and 442868) against the 8H64 protein, CID 443648 (Fig 10E) revealed the highest positive potential at +0.192 a.u. and the highest negative potential at −0.192 a.u. CID 441699 (Fig 10D) had reasonably balanced values of +0.186 and −0.186 a.u., whereas CID 442868 (Fig 10F) demonstrated the lowest potential extremes, varying from +0.09341 to −0.09341 a.u. To evaluate the electrostatic intensity of the examined ligands, the control compound, CID 6249 (Fig 10G), exhibited electrostatic extremes of +0.07489 and −0.07489 a.u.

### 3.7. NBO charge analysis

**Fig 11:**
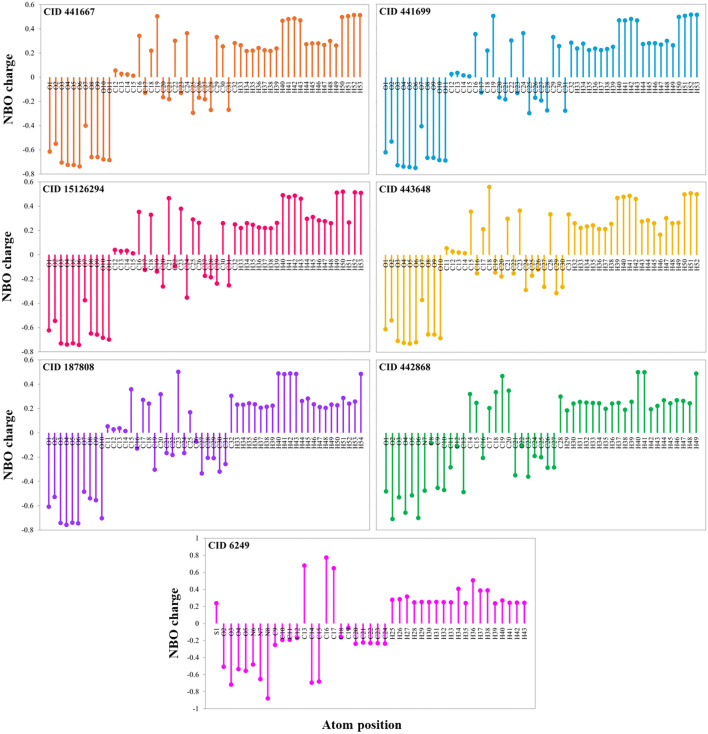
Natural Bond Orbital (NBO) charges plotted against atom positions for the compound CIDs 441667, 15126294, 187808, 441699, 443648, and 442868, alongside the control compound 6249.

Natural bond orbital (NBO) analysis is a practical approach to investigate the charge transmission characteristics and the nature of both intra- and inter-molecular bonding within a molecular system [116]. Charge distribution significantly impacts the stability and chemical reactivity of a chemical bond, as well as the electronic structure and dipole moment polarizability of a molecule [117]. Fig 11 illustrates the NBO charge distribution for six selected compounds along with the control, highlighting the charge polarization among each of the atoms.

Among the three selected compounds (CIDs 441667, CID 15126294, and CID 187808) against the 4CDB protein, CID 15126294 exhibited the highest overall polarization of the three compounds, with oxygen atoms (O1-O11) bearing strongly negative charges around (−0.37 to −0.74 a.u.). In contrast, adjacent carbon atoms, such as C21 and C23, and hydrogen atoms H49-H53 showed correspondingly high positive charges (up to +0.52 a.u.). This significant dipolar character has a high potential for polar and electrostatic interactions with charged or polar residues within the 4CDB binding pocket. Similarly, CID 441667 showed notable negative charges on atoms O3-O6 (−0.72 to −0.74 a.u.) and positive charges on C19 & H52, H53 (up to +0.52 a.u.), whereas CID 187808 revealed notable negative charges on atom O4 (−0.76 a.u.) and positive charges on C23 & H40-43, H54 (up to +0.49 a.u.).

For compounds (CIDs 441699, 443648, and 442868) targeting the 8H64 protein, CID 443648 exhibited the highest unequal charge distribution. Notably, O5 possessed a significant negative charge of −0.73 a.u., whereas atoms C18, C23, and H50-H52 displayed highly positive charges (+0.38 to +0.52 a.u.), indicating considerable potential for electrostatic interactions and hydrogen bonding. Similarly, CID 441699 also exhibited localized polarity, with negatively charged oxygen atoms O3-O6 (−0.72 to −0.74 a.u.) and hydrogens H50-H53 in the + 0.49 to +0.51 a.u. range. Conversely, CID 442868 showed relatively mild charges across atoms, with highly negatively charged O2-O6 (−0.51 to −0.71 a.u.), N7 (−0.48 a.u.) atoms, and positively charged C19 and H40, H41 between +0.47 and +0.50 a.u. CID 6249 exhibited strongly negative NBO charges on atoms O2, O3, N7, O9, C11, and C18 (notably O3 and N7), while C13, C16, and C17 were highly positive, and most hydrogens displayed small positive values, with S1 showing a modestly positive charge.

### 3.8. Molecular dynamics (MD) simulation

This work employed a 100 ns MD simulation to gain a deeper understanding of how the protein’s shape changes in response to a specific drug. Before examining how molecules interact with each other, the 100 ns MD trajectories were used to obtain the final snapshots, which were then evaluated. The Bio3D module of R programming was employed to calculate DCCM and PCA from the MD trajectories [118–120]. The RMSD, RMSF, SASA, rGyr, and H-bond were analyzed to assess the stabilization of the complexes. Additionally, post-simulation MM-GBSA calculations were performed using the Prime MM-GBSA v3.0 tool in Desmond, considering snapshots from the beginning (0 ns) and the end (100 ns) of the simulation.

### 3.9. RMSD analysis of proteins

**Fig 12:**
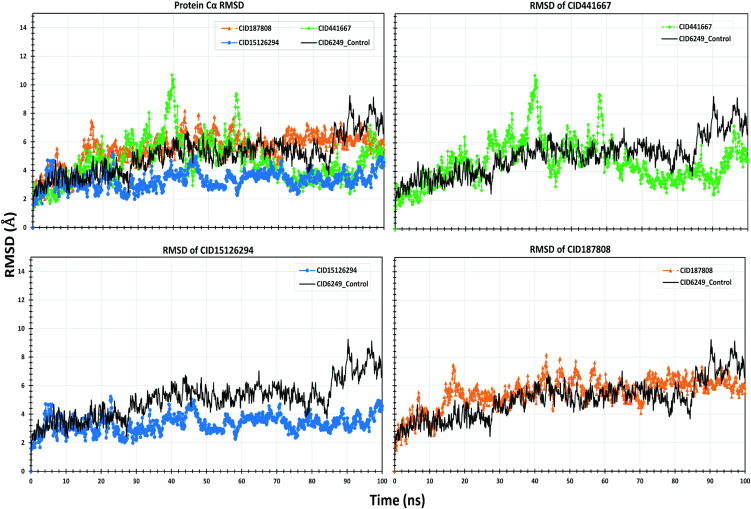
Graphs depicting the MD simulation of the chosen protein-ligand complexes (4CDB), emphasizing the protein Cα RMSD throughout a 100 ns simulation interval. The compounds with CIDs 441667, 15126294, and 187808 were compared with the control compound 6249.

**Fig 13:**
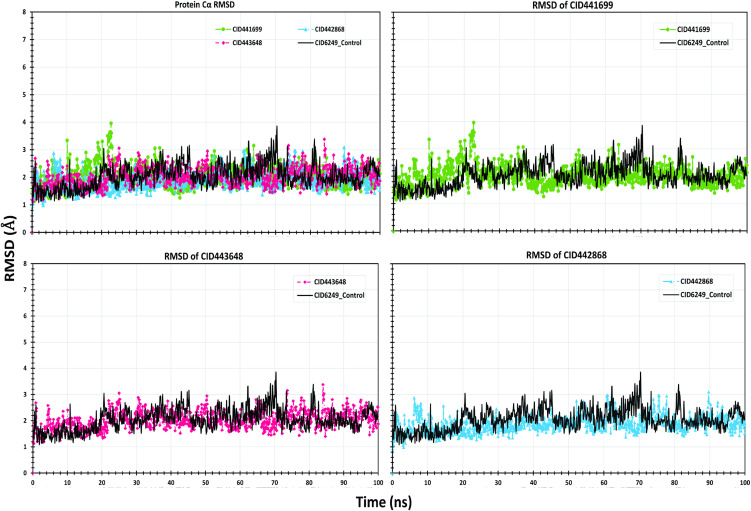
Graphs exhibiting the MD simulation of the chosen protein-ligand complexes (8H64), highlighting the protein Cα RMSD over a 100 ns simulation duration. The compounds CIDs 441699, 443648, and 442868 contrasted with the control compound 6249.

RMSD estimates the average displacement in atoms between frames, which indicates structural variation in the protein-ligand complex over time [121]. A 100 ns MD simulation assessed the conformational variations of the two target proteins (4CDB and 8H64) within the complex of the six selected complexes: CIDs 441667, 15126294, 187808, 441699, 443648, 442868, and 6249 (control), and the corresponding RMSD values were determined. To assess the consistency of the structures of the selected complexes, RMSD values were compared with those of the control drug complex (CID 6249) over a 100 ns simulation duration (Figs 12 and 13, S4 and S5 Files).

In case of 4CDB protein, the greatest RMSD scores of the selected three compounds, CID 441667, CID 15126294, CID 187808, and control (CID 6249), were 10.7 Å, 5.238 Å, 8.17 Å, and 9.23 Å in frame numbers 396, 227, 433 and 903, whereas the smallest were 1.347 Å, 1.559 Å, 1.493 Å, and 1.641 Å in frame numbers 1, 2, 5, and 1, respectively. Only the CID 441667 exhibited notably high RMSD values. Specifically, elevated RMSD values of 10.7, 10.883, 10.371, and 10.217 Å were observed at frames 396, 400, 401, and 399, respectively. The mean RMSD scores for CID 441667, CID 15126294, CID 187808, and control were 4.63 Å, 3.33 Å, 5.52 Å, and 5.03 Å, respectively. The compound CID 441667 had the highest average RMSD value compared to the control & exhibited relatively excessive fluctuation during the 20–60 ns simulation time range. However, it maintained the lowest continuous fluctuation from 60–100 ns, similar to the control (Fig 12). In contrast, the other two selected compounds, CID 15126294 and CID 187808, had the lowest value of average RMSD to the control, exhibiting more consistent fluctuations than the control when binding to the protein. Compared to the control and the other two molecules (CID: 441667 and 187808), CID 15126294 typically maintained consistent fluctuations and displayed excellent results when combined with apoprotein during the simulation period.

Regarding the 8H64 protein, the greatest RMSD scores of the selected three compounds, CID 441699, CID 443648, CID 442868, and control (CID 6249), were 3.96 Å, 3.378 Å, 3.095 Å, and 3.85 Å in frame numbers 227, 840, 897, and 704, while the smallest were 1.147 Å, 1.12 Å, 0.943 Å, and 1.087 Å in frame numbers 8, 3, 1, and 19, accordingly. The average RMSD scores for CID 441699, CID 443648, CID 442868, and control were 2.029 Å, 2.028 Å, 1.88 Å, and 2.05 Å, respectively. Though CID 441699 exhibited relatively high fluctuation between 10 and 30 ns with the control, CID 441699 and CID 443648 have the same average RMSD and the nearest value to the control, exhibiting more consistent fluctuations than the control when bound to the protein (Fig 13). Compared to the control and the other two molecules (CID 441699 and 443648), CID 442868 had the lowest average RMSD value, typically maintains consistent fluctuations, and achieves exceptional outcomes when complexed with apoprotein throughout the simulation period.

### 3.10. Ligand RMSD

Ligand RMSD of the 100 ns simulation was assessed to indicate the ligand’s stability while linked to the protein binding location. The RMSD of the ligand quantifies the mean difference between atomic positions in its current conformation and its original conformation. A lower RMSD value denotes that the ligand’s structure is closely aligned with its reference state, signifying enhanced stability and less movement.

**Fig 14:**
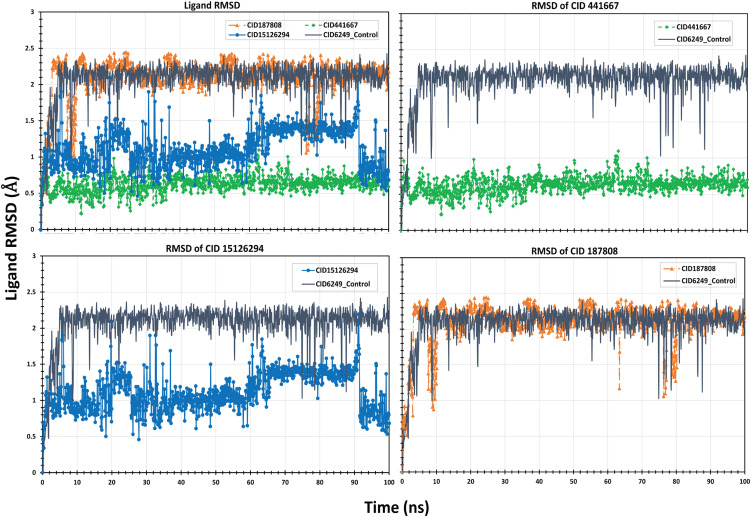
Presenting the RMSD values derived from the ligand atoms against the 4CDB protein, including CIDs 441667, 15126294, and 187808, along with the control 6249, throughout a 100 ns simulation period.

Regarding the 4CDB protein, the three chosen compounds (CIDs 441667, 15126294, and 187808), along with the control (CID 6249), exhibited estimated average values of 0.63 Å, 1.12 Å, 2.095 Å, and 2.073 Å, respectively, as displayed in Fig 14 and S6 File. This study indicates that CID 441667 and CID 15126294 exhibited significant deviation from the control compound. Still, CID 187808 aligned with the control compound throughout the entire simulation period.

**Fig 15:**
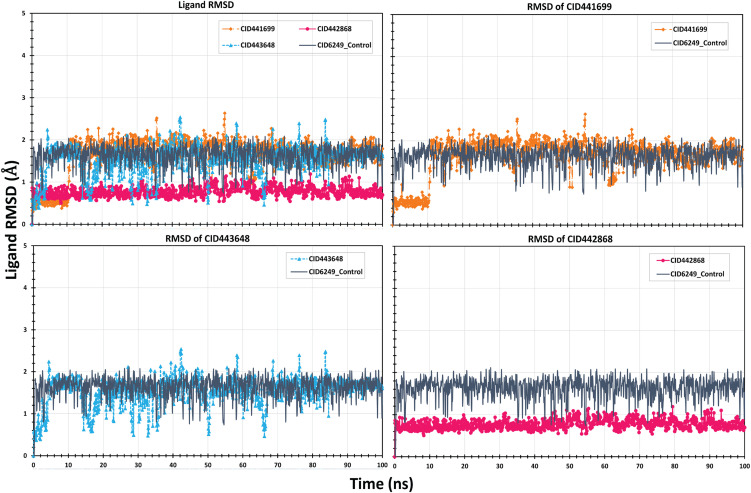
Presenting the RMSD values derived from the ligand atoms against the 8H64 protein, including CIDs 441699, 443648, and 442868, along with control 6249, during a 100 ns simulation period.

In case of the 8H64 protein, the three selected compounds (CID 441699, CID 443648, and CID 442868) along with the control (CID 6249) displayed approximate average values of 1.64 Å, 1.54 Å, 0.772 Å, and 1.63 Å, accordingly, as depicted in Fig 15 and S7 File. This study indicates that CID 441699 displayed relatively extensive fluctuation within the 0–10 ns simulation time frame, whereas it sustained the lowest steady fluctuation from 10 to 100 ns, compared to the control. In contrast, CID 442868 exhibited a significant deviation from the control drug complexes, whereas CID 443648 aligned with the control compound-complex throughout the entire 100 ns simulation duration.

### 3.11. RMSF analysis

**Fig 16:**
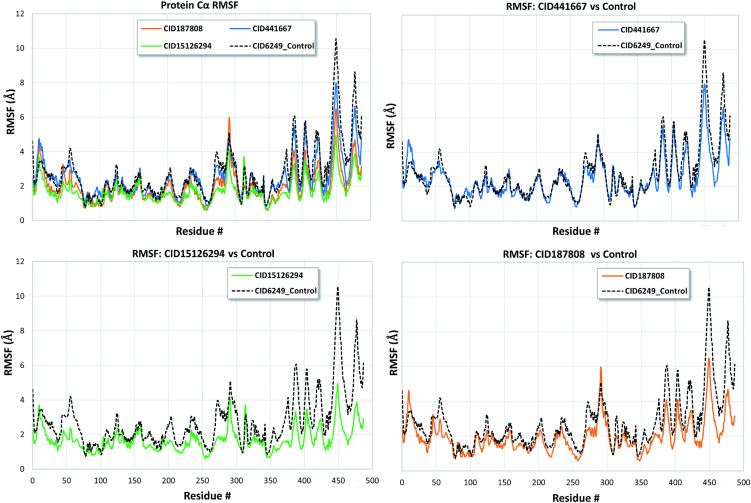
RMSF values were depicted for the selected compounds (CIDs 441667, 15126294, and 187808) and control 6249 against the 4CDB protein retrieved from the complex system’s C α atoms.

The RMSF enables the assessment of macromolecular diversity and static conditions, offering insights into the regional conformational alterations of amino acid residues [122]. In 4CDB protein, the RMSF values for the three selected compounds (CID 441667, CID 15126294, and CID 187808) and control (CID 6249) were as follows: the lowest values were 0.784 Å, 0.604 Å, 0.579 Å, and 0.743 Å, respectively at residue positions PRO 384, ILE 293, TYR 387, and VAL 117; the highest values were 7.977 Å, 4.949 Å, 6.487 Å, and 10.552 Å, accordingly at residue positions ALA 488, ALA 488, ALA 488, and ALA 488, illustrated in Fig 16 and S8 File. The average RMSF values for the three chosen compounds and the control were 2.5 Å, 1.716 Å, 1.994 Å, and 2.756 Å, accordingly.

**Fig 17:**
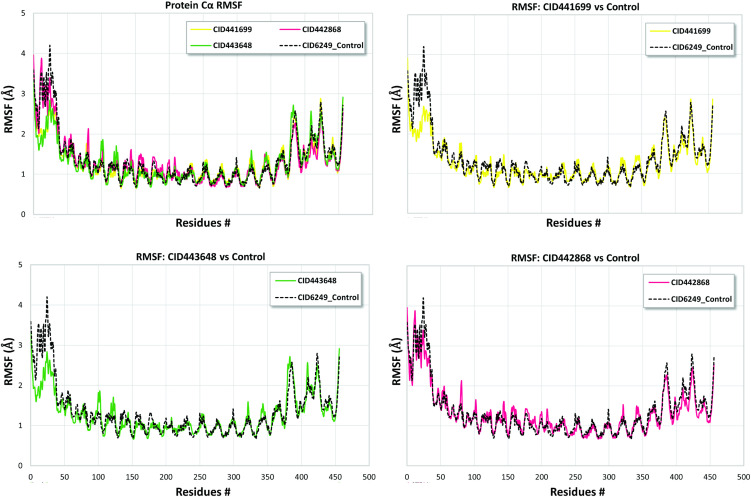
RMSF values were illustrated for the selected compounds (CIDs 441699, 443648, and 442868) and control 6249 next to the 4CDB protein retrieved from the complex system’s C α atoms.

In 8H64 protein, the RMSF scores for the three selected compounds CIDs 441699, 443648, and 442868 and 6249 (control) were as follows: the lowest values were 0.65 Å, 0.66 Å, 0.668 Å, and 0.659 Å, respectively at residue positions LEU 169, SER 192, LEU 324, and VAL 373; the highest values were 3.915 Å, 3.354 Å, 3.958 Å, and 4.199 Å, accordingly at residue positions GLN 40, GLN 40, GLN 40, and THR 64, as represented in Fig 17 and S9 File. The average RMSF values for the three chosen compounds and the control were 1.278 Å, 1.276 Å, 1.324 Å, and 1.354 Å, accordingly.

### 3.12. Radius of gyration (rGyr) analysis

In a protein-ligand complex, the angular distribution of atoms relative to the axis is denoted as the radius of gyration (rGyr) [74]. The rGyr, the square of the radial distance from the center of mass of protein-ligand complexes, measures both the compactness and movement of a protein at its terminal and is the typical alteration in protein-ligand compactness resulting from macromolecular structural dynamics [123]. A higher rGyr score denotes loosely packed protein molecules, while a lower rGyr value implies firmly packed protein structures. [124].

**Fig 18:**
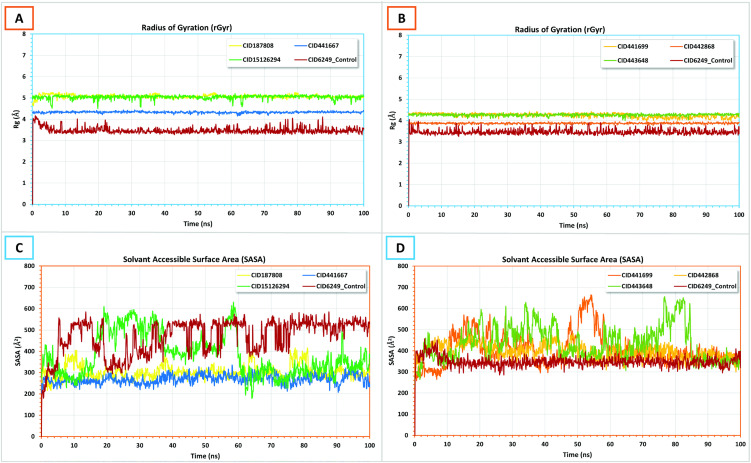
Graphs **(A)** and **(B)** represent radius of gyration (rGyr) values, whereas Graphs **(C)** and **(D)** represent Solvent accessible surface area (SASA) values regarding 4CDB & 8H64 protein, extruded from the complex structures of ligand atoms. All of the values were represented by CID 441667 (blue), CID 15126294 (green), CID 187808 (yellow), CID 441699 (orange), CID 443648 (light green), CID 442868 (gold), and CID 6249 (control) (dark red).

Regarding the 4CDB protein, Fig 18A and S6 File showed that the average rGyr values for the CIDs 441667, 15126294, 187808, and 6249 (control) were calculated to be 4.326 Å, 5.042 Å, 5.071 Å, and 3.472 Å, accordingly. The rGyr results indicate that all selected compounds displayed a radial distance close to the control compound and suggest that the protein’s active site undergoes modest structural modification upon binding to the selected compounds. CIDs 441667, 15126294, and 187808 all had higher rGyr values, which means they are loosely packed with the protein, other than the CID 6249, which worked as a control and had a lower rGyr value. CID 15126294 and CID 187808 had relatively similar rGyr values and overlapped with each other, whereas CID 15126294 showed some lower fluctuations during the simulation period. The control compound exhibited the highest fluctuations from 8 to 24 ns and 64–88 ns, where CID 441667 had a lower rGyr value compared to the other two selected compounds and did not fluctuate at all during the simulation time.

Regarding the 8H64 protein, Fig 18B and S7 File showed that the average rGyr scores for the CIDs 441699, 443648, 442868, and 6249 (control) were calculated to be 4.246 Å, 4.266 Å, 3.873 Å, and 3.461 Å, respectively. According to the results, CID 442868 displayed relatively similar rGyr values compared to the control. These results indicate that all selected compounds exhibited a lower radial distance compared to the control, suggesting that the protein’s active site remains structurally stable upon binding to these compounds. The control compound exhibited fluctuations, whereas CID 441699 & CID 443648 overlapped with each other and fluctuated the least during the simulation time.

### 3.13. Solvent accessible surface area (SASA) analysis

SASA is the outermost area at which a ligand or protein interacts with solvent molecules, and it correlates with the solvent-complex interactions occurring over the simulation study [125,126]. The SASA value is utilized to assess the extent and importance of the protein conformation that transpires upon ligand-receptor binding [127]. A higher SASA value denotes a dispersed protein structure, whereas a lower SASA score signifies a compact structure [128]. Therefore, the minimal and maximal SASA values of the 4CDB protein were calculated as (202.701–346.414) Å^2^ for CID 441667 in frame numbers 1 & 955, (179.837–630.397) Å^2^ for CID 15126294 in frame numbers 641 & 585, (215.844–419.267) Å^2^ for CID 187808 in the frame numbers 19 & 633 and (180.122–585.308) Å^2^ for CID 6249 (control) in frame numbers 5 & 874, respectively, displayed in Fig 18C and S6 File. The average SASA values of the key compounds were 269 Å^2^, 382.81 Å^2^, and 302.69 Å^2^; in contrast, the control had an SASA value of 469.69 Å^2^, indicating decreased exposure of targeted protein amino acids to the active site when complexed with the leading compounds.

Furthermore, the minimum and maximum SASA values of the 8H64 protein were evaluated as (251.696–664.511) Å^2^ for CID 441699 in frame numbers 0 & 541, (232.126–655.534) Å^2^ for CID 443648 in frame numbers 0 & 767, (247.938–482.72) Å^2^ for CID 442868 in the frame numbers 0 & 165 and (280.74–457.198) Å^2^ for CID 6249 (control) in frame numbers 0 & 29, accordingly, depicted in Fig 18D and S7 File. The average SASA values of the key compounds were 407.13 Å^2^, 433.37 Å^2^, and 397.42 Å^2^; in contrast, the control exhibited SASA scores of 348.47 Å^2^, as well. The results showed that CID 442868 had an average SASA value nearly equal to the value of the control compound.

### 3.14. Assessment of protein-ligand (P-L) interactions

Protein-ligand (P-L) interactions have been thoroughly used to examine the molecular links between bound ligands and residues in proteins’ active sites. The simulation interactions diagram (SID) was used to study a protein’s complex structure, ligands, and intermolecular connections. The contact between the protein and the selected compounds is predicated upon several parameters, including non-covalent bonds (hydrophobic bond), hydrogen bonds, water bridge bonds, and ionic bonds.

**Fig 19:**
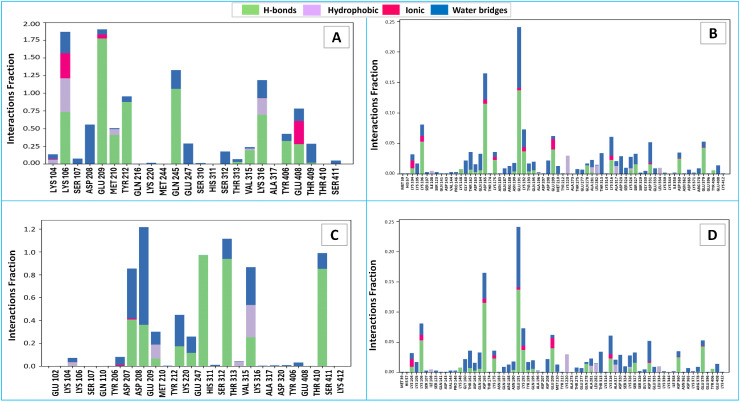
The structured bar charts present the protein-ligand interactions observed over the 100 ns simulation. This figure illustrates the interaction of the Internalin A (4CDB) protein with CIDs 441667 **(A)**, 15126294 **(B)**, 187808 **(C)**, and 6249 (control) **(D)**.

Therefore, the interaction between the 4CDB protein and the selected compounds, CIDs 441667, 15126294, and 187808, along with 6249 (control), has been studied and illustrated in **Fig 19**. CID 441667 produced numerous interactions at the residues LYS 104, LYS 106, GLU 209, MET 210, VAL 315, LYS 316, and GLU 408, with interaction fractions (IF) of 0.15, 1.90, 1.95, 0.51, 0.25, 1.20, and 0.77, respectively, as seen in **Fig 19A**. The durable, unique connection occurred during the simulation as the same subtype continuously interacted with the ligand. In **Fig 19B**, several interactions within the compound CID 15126294 and the residues LYS 106 (0.18), GLN 110 (0.14), ILE 204 (0.13), TYR 206 (0.38), MET 210 (0.04), LYS 220 (0.07), LYS 314 (0.02), LYS 316 (0.15), ASP 320 (1.64), VAL 323 (0.32), LYS 326 (0.1), SER 329 (1.85), LEU 370 (0.21), LYS 371 (0.05) and GLU 408 (1.18) were generated, according to the period of the simulation. In **Fig 19C**, another lead compound CID 187808 created multiple interactions with the residues LYS 106 (0.8), ASP 208 (0.85), MET 210 (0.33), and LYS 316 (0.88), where numerous interactions were made by the control compound at residues LYS 104 (0.036), LYS 106 (0.08), ASP 165 (0.168), LYS 175 (0.04), GLU 191 (0.24), LYS 192 (0.075), GLU 209 (0.065), ALA 281 (0.025), LYS 316 (0.06), LYS 326 (0.04), ASP 331 (0.052), ASP 360 (0.038) and GLU 379 (0.054), all of which were maintained according to the simulation duration, presented in **Fig 19D**. The compound CID 15126294 revealed stronger bonding with the apoprotein than the other two lead compounds (CIDs 441667 and 187808) and the control.

**Fig 20:**
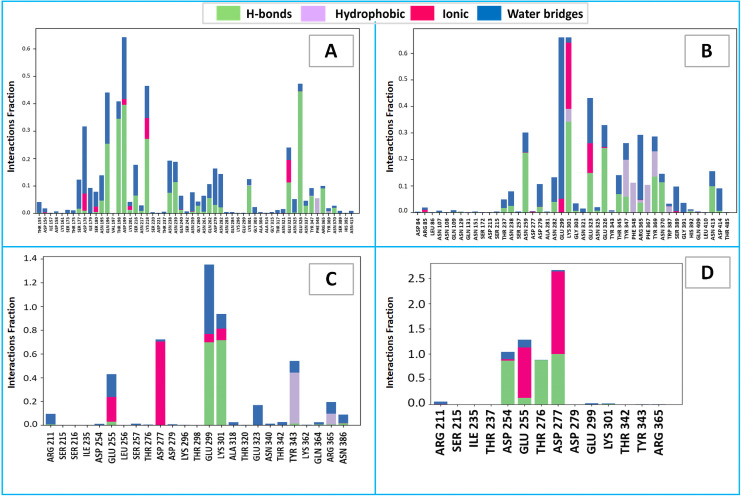
The structured bar charts present the protein-ligand interactions observed over the 100 ns simulation. This figure illustrates the interaction of the Listeriolysin O (8H64) protein with CIDs 441699 **(A)**, 443648 **(B)**, 442868 **(C)**, and 6249 (control) **(D)**.

Furthermore, the interaction between the 8H64 protein and the selected compounds (CIDs 441699, 443648, 442868, and the control 6249) has been analyzed and is represented in **Fig 20**. CID 441699 exhibited numerous interactions at the residues ASP 156, ASP 178, SER 180, ASP 199, LYS 201, LYS 218, GLN 240, ASN 259, LYS 301, GLU 323, GLU 326, TYR 347, and TYR 369 with interaction fractions (IF) of 0.3, 0.32, 0.08, 0.8, 0.06, 0.47, 0.07, 0.09, 0.12, 0.24, 0.49, 0.1, and 0.02, accordingly, as seen in **Fig 20A**. The firm, distinctive interaction transpired during the simulation as the identical subtype persistently engaged with the ligand. In **Fig 20B**, several interactions between the compound CID 443648 and the residues ARG 85 (0.02), ASN 259 (0.3), ASP 279 (0.11), ALA 281 (0.01), LYS 301 (0.68), GLU 323 (0.43), GLU 326 (0.34), TYR 343 (0.01), TYR 347 (0.28), ARG 365 (0.3), TYR 369 (0.29), TRP 387 (0.04), and HIS 392 (0.02) were generated, according to the period of the simulation. In **Fig 20C**, another lead compound CID 442868 created multiple interactions with the residues GLU 255 (0.43), GLU 299 (1.36), LYS 301 (0.97), TYR 343 (0.58), ARG 365 (0.2), and ASN 386 (0.1) where numerous interactions were made by the control compound at residues ARG 211 (0.01), ASP 254 (1.1), GLU 255 (1.3), and ASP 277 (2.7) all of which were sustained according to the simulation, presented in **Fig 20D**. The compounds CID 441699 and CID 443648 revealed stronger bonding with the apoprotein than the control.

### 3.15. Assessment of ligand-protein interactions

**Fig 21:**
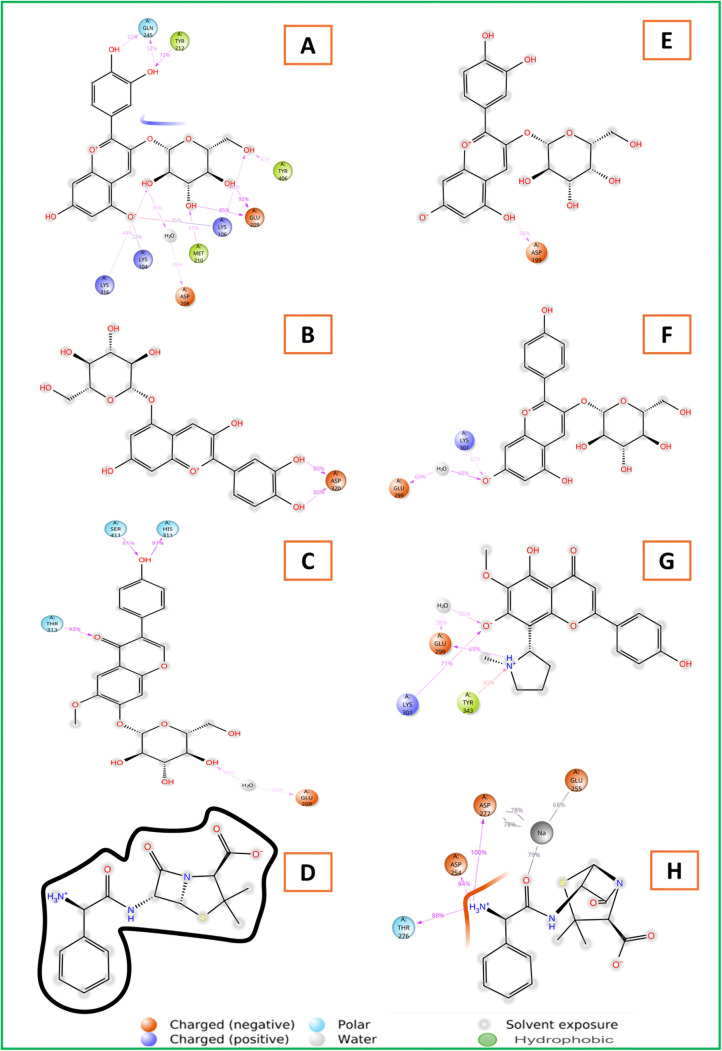
The graph illustrates the interactions among the protein 4CDB and ligands CIDs 441667 **(A)**, 15126294 **(B)**, 187808 **(C)**, and 6249 (control) **(D)**, as well as the interactions between the protein 8H64 and ligands CIDs 441699 **(E)**, 443648 **(F)**, 442868 **(G)**, and 6249 (control) **(H)**, exhibiting the structural symmetry of the ligands interacting with the native protein.

Ligand-protein interaction is the examination of the binding behavior of tiny molecules, such as ligands, during efficient drug development. The simulation interactions diagram (SID) helped to understand and visualize several interactions between apoprotein (4CDB) and the complex of three selected ligands, CIDs 441667, 15126294, 187808, and control ligand 6249, throughout the trajectory and extracted interaction data, shown in Fig 21. Throughout the simulation period, these compounds showed a significant number of identical subtype interactions between the ligands CID 441667, CID 187808, and the apoprotein, with each exhibiting more than two interactions. In Fig 21A, the CID 441667 ligand structure showed nine amino acid residues, which were GLY 245, TYR 212, TYR 406, GLU 209, LYS 104, LYS 106, LYS 316, MET 210, ASP 208, and one water molecule was bonded to it, respectively. Likewise, ligand CID 187808 (Fig 21B) bound to the amino acid residues SER 411, HIS 311, THR 313, GLU 209, as well as a water molecule, where ligand CID 15126294 (Fig 21C) bound with only one amino acid residue, ASP 320, accordingly.

Furthermore, the interaction between the 8H64 and the complex of three selected ligands, CID 441699, CID 443648, CID 442868, and the control ligand (CID 6249), had been observed to have protein interactions across the entire SID, shown in Fig 21. The compounds CID 443648, CID 442868, and control produced several interactions during the simulation, with the specific interaction sustained by several contacts of the identical subtype with the ligand. In Fig 21G, CID 442868 ligand structure showed three amino acid residues, which were GLU 299, LYS 301, TYR 343 and one water molecule was bonded to it, as well as, in Fig 21H, ligand CID 6249 (control) showed four amino acid residues, which were GLU 255, ASP 277, ASP 254, THR 276 and one sodium molecule is bonded to it, respectively. Likewise, ligand CID 443648 (Fig 21F) bound to the amino acid residues GLU 299 and LYS 301, as well as a water molecule, whereas 441699 (Fig 21E) bound with only one amino acid residue, ASP 199. Notably, the control exhibited fewer binding contacts, suggesting weaker stabilization within the active site of the protein.

### 3.16. Post-simulation MM-GBSA analysis

**Fig 22:**
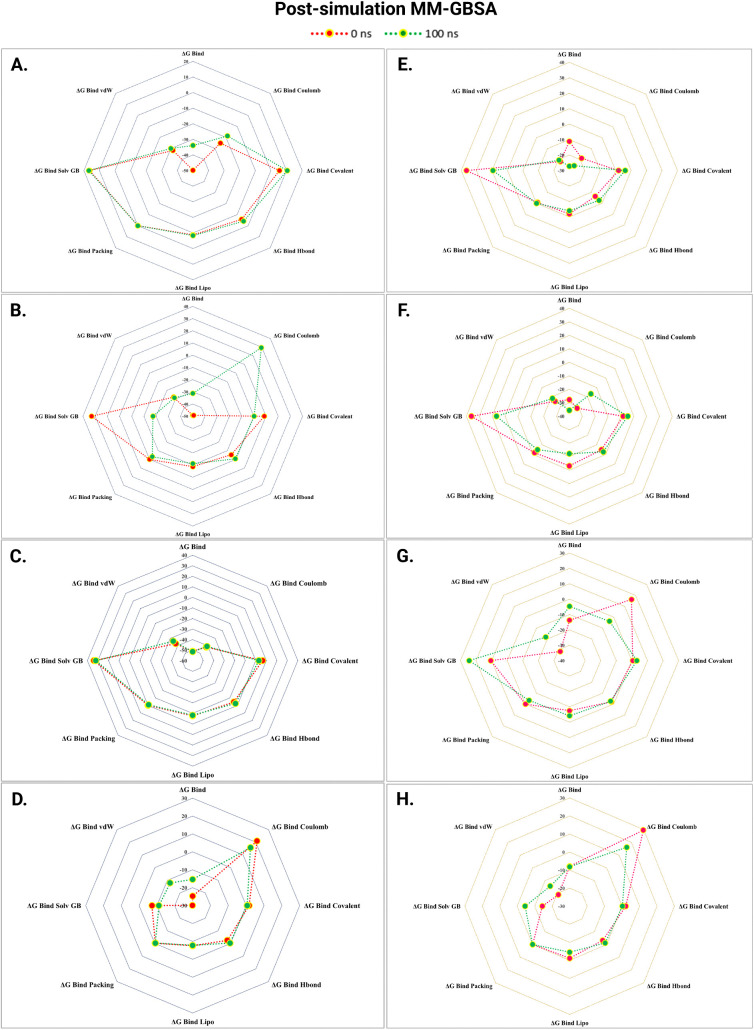
Post-simulation MM-GBSA analysis of the compounds CIDs 441667 **(A)**, 15126294 **(B)**, 187808 **(C)**, and 6249 (control) **(D)** in complex with the 4CDB protein, along with CIDs 441699 **(E)**, 443648 **(F)**, 442868 **(G)**, and 6249 (control) **(H)** in complex with the 8H64 protein.

The MM-GBSA analysis was conducted on the 4CDB and 8H64 complexes over a 100 ns trajectory to assess the binding stability and thermodynamic characteristics of the selected compounds (Fig 22, S10 and S11 Files). The binding affinity for the 4CDB protein-ligand complex remained significantly superior to the control (CID 6249) throughout the simulation. CID 187808 exhibited the highest stability, with ΔG Bind values shifting slightly from −51.09 kcal/mol at 0 ns to −51.47 kcal/mol at 100 ns (Fig 22C). Similarly, CID 441667 showed a decrease in negative binding free energy (ΔG Bind), moving from −49.79 kcal/mol to −33.91 kcal/mol (Fig 22A), while CID 15126294 shifted from −49.33 kcal/mol to −31.29 kcal/mol (Fig 22B), respectively. Despite these fluctuations, all three candidates significantly outperformed CID 6249 (control), which decreased from an initial −24.73 kcal/mol to −15.38 kcal/mol (Fig 22D).

Regarding the 8H64 protein-ligand complex, CID 443648 displayed the most significant improvement in affinity, with negative binding free energy (ΔG Bind) increasing from −27.73 kcal/mol at 0 ns to −35.62 kcal/mol at 100 ns, suggesting an induced-fit optimization (Fig 22F). Additionally, CID 441699 showed favorable progression, moving from ΔG Bind values of −11.13 kcal/mol to −27.22 kcal/mol (Fig 22E), and CID 442868 exhibited a loss of stability, with its ΔG Bind values reduced from −13.67 kcal/mol to −4.65 kcal/mol by the end of the simulation (Fig 22G), respectively. In contrast, the control exhibited a lower energy value with minimal change, transitioning slightly from −7.87 kcal/mol to −8.11 kcal/mol (Fig 22H).

### 3.17. PCA analysis

**Fig 23:**
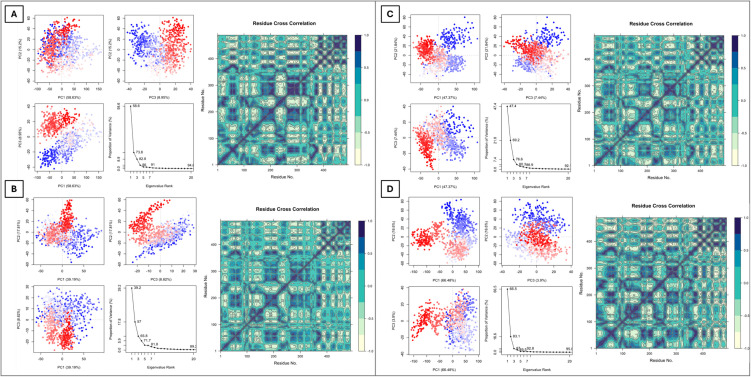
The PCA results (on the left) show eigenvalues over the proportion of variance for PC1, PC2, and PC3, with each region shown separately. The DCCM plots (on the right) show cross-correlation patterns, delineated by sea green and blue, respectively, indicating negative and positive. Here, the CIDs **(A)** 441667, **(B)** 15126294, **(C)** 187808, and **(D)** 6249 (control) are all against the 4CDB protein.

The conformational landscapes of the protein-ligand complexes were investigated by using Principal Component Analysis (PCA) on their molecular dynamic trajectories [77,113,129]. PCA was conducted to assess the structural dynamics of the protein–ligand complexes, with the control complex. Considering the protein 4CDB, results indicated that the first principal component (PC1) accounted for the highest proportion of variance in each system, with values of 58.63%, 39.19%, 47.37%, and 66.48% for complexes 4CDB-CID 441667 (Fig 23A), 4CDB-CID 15126294 (Fig 23B), 4CDB-CID 187808 (Fig 23C), and the control (4CDB-CID 6249) (Fig 23D), respectively. Additionally, PC2 contributed 15.23%, 17.81%, 21.54%, and 16.61% to the total variance, while PC3 explained 8.96%, 8.82%, 7.44%, and 3.9% for complex 4CDB-CID 441667, 4CDB-CID 15126294, 4CDB-CID 187808, and the control, as well. The cumulative variance represented by the initial three principal components reached 82.6% for 4CDB-CID 441667, 65.6% for 4CDB-CID 15126294, 76.3% for 4CDB-CID 187808, and 83.1% for the control, as illustrated in the scree plots in Fig 23.

**Fig 24:**
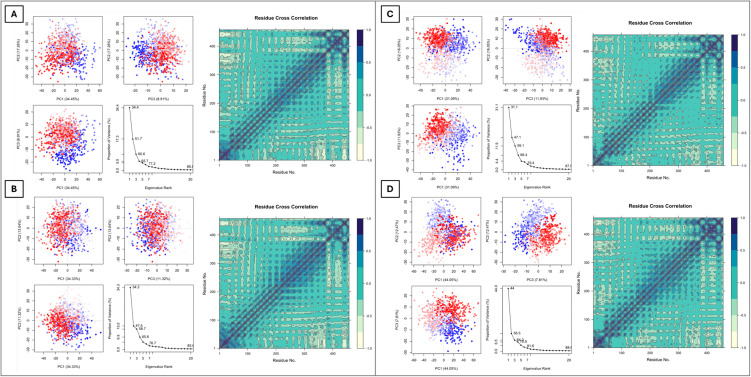
The PCA results (on the left) show eigenvalues over the proportion of variance for PC1, PC2, and PC3, with each region shown separately. The DCCM plots (on the right) show cross-correlation patterns, delineated by sea green and blue, respectively, indicating negative and positive. Here, the CIDs **(A)** 441699, **(B)** 443648, **(C)** 442868, and **(D)** 6249 (control) are all directed toward the 8H64 protein.

Regarding the protein 8H64, findings revealed that the first principal component (PC1) captured the greatest proportion of variance across all datasets: 34.45% for complex 8H64-CID 441699 (Fig 24A), 34.33% for complex 8H64-CID 443648 (Fig 24B), 31.09% for complex 8H64-CID 442868 (Fig 24C), and 44.09% for the control (8H64-CID 6249) (Fig 24D), correspondingly. Moreover, PC2 accounted for 17.26% in 8H64-CID 441699, 13.25% in 8H64-CID 443648, 16.95% in 8H64-CID 442868, and 12.74% in the control, whereas PC3 explained 9.13% for 8H64-CID 441699, 11.32% for 8H64-CID 443648, 11.93% for 8H64-CID 442868, and 7.81% for the control, as well. In Fig 24, the scree plots indicated that the cumulative variance explained by the first three PCs reached 60.8% (8H64-CID 443699), 58.9% (8H64-CID 443648), 59.9% (8H64-CID 442868), and 64.6% (8H64-CID 6249), indicating that a significant portion of the system’s motions was described by these PCs. The eigenvalue distributions supported that the majority of the system’s dynamic variance was concentrated in the first few principal components for all complexes. The PCA scatter plots revealed distinct clustering and separation patterns among the complexes, indicating differences in structural flexibility and motions induced by their respective ligands.

### 3.18. DCCM analysis

Dynamic Cross-Correlation Matrix (DCCM) analysis was performed to assess the residue-residue motion correlations within the protein 4CDB in complex with three lead compounds (CIDs 441667, 15126294, and 187808) and within the protein 8H64 in complex with three lead compounds (CIDs 441699, 443648, and 442868), as well as the control (CID 6249), accordingly. In Figs 23 and 24, the DCCM heatmaps, representing cross-correlation coefficients between residue motions, reveal distinct dynamic patterns upon ligand binding. When bound to the lead compounds, the 4CDB protein exhibited altered correlation networks, demonstrating that ligand interaction significantly modulates internal protein dynamics (Fig 23). Among the complexes, lead compound CID 15126294 (Fig 23B) induced the most extensive and intense correlation across several regions, as observed by more prominent, contiguous blue regions, suggesting a reinforcement of cooperative residue dynamics and potential stabilization of key motion pathways. Compounds CID 441667 (Fig 23A) and CID 187808 (Fig 23C) also altered the native correlation landscape, but their effects were less pronounced and more localized compared to CID 15126294, with compound CID 187808 producing a somewhat fragmented interaction pattern.

Regarding the 8H64 protein, the DCCM plots (Fig 24) displayed the extent and pattern of correlated (blue) and anticorrelated (yellow) motions between all residue pairs during molecular dynamics simulations. The control (8H64-CID 6249) (Fig 24D) complex demonstrated widespread moderate correlation across multiple regions, indicating intrinsic dynamic connectivity of the apo-protein. Notably, complexes with lead compounds 8H64-CID 442699 (Fig 24A), 8H64-CID 443648 (Fig 24B), and 8H64-CID 442868 (Fig 24C) displayed altered correlation patterns relative to the control, suggesting a significant impact of ligand binding on internal protein dynamics. Among these, complex 8H64-CID 443648 exhibited the most pronounced and extensive correlated motion, reflected by larger and darker blue regions on the map, particularly along specific residue segments, suggesting enhanced cooperative dynamics induced by the ligand. In contrast, complexes 8H64-CID 442699 and 8H64-CID 442868 showed modified but less extensive correlation networks compared to 8H64-CID 443648, with 8H64-CID 442868 displaying a more dispersed correlation profile.

## 4. Discussion

*Listeria monocytogenes* is a pathogenic bacterium responsible for severe food-borne infections in high-risk populations, with limited treatment options. To explore alternatives, flavonoids, a plant-derived secondary metabolite with antimicrobial potential, were evaluated using computer-aided drug design. Six flavonoid candidates were evaluated alongside ampicillin for drug-likeness and toxicity, considering parameters such as rotatable bonds, hydrogen bonding, lipophilicity, solubility, GI absorption, and compliance with Lipinski’s rule of five. For 4CDB, CID 441667 and 187808 exhibited optimal properties, with high GI absorption and no rule violations. Similarly, for 8H64, CID 442868 and 441699 showed favorable profiles. All six compounds surpassed ampicillin in drug-likeness, with low toxicity and high LD_50_ values. Among them, CID 441667, 441699, 15126294, and 443648 emerged as the most promising leads, combining robust oral bioavailability with consistently lower toxicity scores, emphasizing their potential as favorable therapeutic agents against *L. monocytogenes*. The superior binding affinities of the selected flavonoids compared to the reference drug ampicillin further validate the robustness of the docking-based screening approach.

Analysis of protein-ligand interactions revealed that in 4CDB, CID 15126294 formed the highest number of hydrogen bonds, while CID 441667 and 187808 showed four interactions each, indicating strong complex stability compared to the control, which formed only one. For 8H64, CID 441699 and 443648 exhibited strong, specific interactions comparable to the control but with superior drug-like properties, highlighting their potential as viable therapeutic candidates.

The electrical properties of drug candidates were evaluated using QM calculations, revealing that a diminished HOMO-LUMO gap signified enhanced chemical reactivity and biological activity. CIDs 441667 and 15126294 exhibited superior performance compared to the control, demonstrating the lowest energy gaps, chemical hardness, and softness for 4CDB. In comparison to the control lead, the minimal HOMO-LUMO gaps, hardness, and softness of CIDs 443648 and 441699 for 8H64 suggested enhanced reactivity and therapeutic potential. MEP analysis identified electrophilic and nucleophilic attack regions to assess molecule reactivity and interaction potential. For 4CDB, CIDs 15126294 and 441667 had higher charge distribution, suggesting stronger polar interactions, whereas CID 187808 was marginally superior to the control. CID 443648, 441699, and 442868 had the maximum electrostatic intensity in the case of 8H64, exceeding the control. Overall, CIDs 15126294, 441667, 443648, and 441699 had the most favorable profiles, indicating more substantial and specific molecular interactions. NBO analysis revealed that the chosen compounds exhibit enhanced charge polarization relative to the control. Focusing on 4CDB, CID 15126294 had the most pronounced dipolar characteristics, with significantly negative oxygen atoms (O1-O11) and positively charged neighboring atoms (C21, C23, H49-H53), indicating augmented electrostatic and polar interactions; CID 441667 and 187808 displayed mild polarization. In 8H64, CID 443648 exhibited the most significant charge difference, followed by CID 441699 and CID 442868, which had modest impacts.

Molecular dynamics simulations, conducted over a 100 ns timescale, were employed to assess protein–ligand complex stability, providing significant insights into their dynamics and conformational behavior. RMSD analysis showed CID 15126294 and CID 187808 as most stable for 4CDB, while CID 442868 and CID 443648 were most stable for 8H64. Ligand RMSD confirmed CID 441667 and CID 15126294 as most stable for 4CDB, and CID 442868 and CID 443648 for 8H64. RMSF further highlighted CID 15126294 and CID 187808, reducing flexibility in 4CDB, whereas CID 443648 and CID 441699 stabilized 8H64, marking them as strong lead candidates.

Radius of gyration analysis indicated CID 441667 and CID 15126294 created the most compact 4CDB complexes, while CID 442868 and 443648 stabilized the 8H64. CID 441667 and 187808 were the most compact for 4CDB, and 442868 and 441699 for 8H64, according to SASA. The interactions showed that CID 15126294 and CID 441667 firmly bound to important 4CDB residues, whereas CID 441699 and CID 443648 were most consistent for 8H64, making them leading candidates. Nevertheless, the MM‑GBSA analysis revealed that, compared to the control CID 6249, CID 441667 and CID 187808 formed more stable complexes with 4CDB, while CID 441699 and CID 443648 exhibited improved binding with 8H64, indicating enhanced target engagement and thermodynamic stability for these selected candidates.

PCA demonstrated stable 4CDB and 8H64 complexes with most conformational dynamics in the first three main components. In 4CDB, the variance distributions of 4CDB-CID 441667 (cumulative variance about 82.6%) and 4CDB-CID 187808 (cumulative variance about 76.3%) complexes were identical to the control (4CDB-CID 6249) complex (cumulative variance about 83.1%), showing steady binding and no structural disturbance. 8H64-CID 441699 and 8H64-CID 442868 complexes had similar clustering patterns and cumulative variance of about 60.8% and 59.9%, respectively, compared to the control complex (cumulative variance of about 64.6%) in 8H64, suggesting advantageous dynamic features. These structurally stable molecules may be attractive study subjects. DCCM indicated that ligand binding significantly changed 4CDB and 8H64 protein dynamics. CID 15126294 produced 4CDB the most correlated motions, implying greater cooperative dynamics and structural stabilization, while CID 441667 promoted ordered but restricted correlations. CID 443648 exhibited the largest associated residue motion in 8H64, indicating strong dynamic coupling, followed by CID 442868, which had moderate but positive alterations. These compounds affected protein flexibility and dynamic communication, making them promising.

This study proposes a therapy strategy against listeriosis by focusing on the inhibition of two key proteins, 4CDB and 8H64, using specifically discovered bioactive flavonoid lead compounds. This approach aimed to specifically disrupt protein activity that is critical to the pathogen’s survival, even though the bulk of existing treatments rely on broad-spectrum antibiotics. Additional study evidence on natural-product inhibitors of *L. monocytogenes*, including investigations of plant phenolics, berberine derivatives, essential oil constituents, and catechin-based antibiofilm compounds, further supports the topic [130–133]. These comparison analogies improve the contextual depth of the computational outcomes and more accurately place flavonoid-mediated inhibition within the larger framework of natural antibacterial research. These substances had the potential to halt the progression of listeriosis since they had been exhibited using molecular docking, molecular dynamics simulation, PCA, and DCCM analysis to significantly alter protein behavior. This investigation is novel as it evaluates the inhibitory potential of flavonoids against *L. monocytogenes* proteins 4CDB and 8H64, an area with limited prior study using such a comprehensive flavonoid dataset. This study thereby establishes a distinctive foundation for the development of flavonoid-based anti-listerial therapeutics, a new step toward more effective and precise anti-listerial medications. Nevertheless, computational techniques can only be used to estimate the effectiveness of drug candidates in a fictitious environment. Differential results were predicted by examining the proposed compounds according to various dimensions. Therefore, to validate this discovery, more model organism experiments are required. These findings might encourage researchers to trait wet lab testing for future investigations.

## 5. Conclusion

*Listeria monocytogenes* remains a serious foodborne pathogen responsible for listeriosis, including pregnancy-associated complications, neonatal infections, and central nervous system disorders such as meningoencephalitis. The bacterium’s ability to invade host cells, survive intracellularly, and spread between cells is mediated by key virulence factors, *Internalin A (InlA)* and *Listeriolysin O (LLO)*. This study demonstrates that plant-derived flavonoids can effectively target these factors, using an integrated *in-silico* approach. Among 1,254 screened compounds, Cyanidin 3-glucoside (CID 441667) and Cyanidin 5-O-glucoside (CID 15126294) emerged as promising inhibitors of *InlA*, while Cyanidin 3-O-galactoside (CID 441699) and Pelargonidin 3-glucoside ion (CID 443648) were identified as potential *LLO* inhibitors. Molecular docking, admet profiling, QM calculations, and molecular dynamics simulations predicted the stability, drug-likeness, and binding affinity of these compounds against their respective targets, followed by post-simulation MM-GBSA, PCA and DCCM analysis. These findings highlight promising flavonoid candidates that warrant further *in vitro* and *in vivo* validation.

## Supporting information

S1 File ADME. (DOCX)

S2 File Toxicity. (DOCX)

S3 File Docking interactions. (DOCX)

S4 File Protein RMSD of 4CDB. (XLSX)

S5 File Protein RMSD of 8H64. (XLSX)

S6 File Ligand RMSD, Rg, SASA of 4CDB. (XLSX)

S7 File Ligand RMSD, Rg, SASA of 8H64. (XLSX)

S8 File Protein RMSF of 4CDB. (XLSX)

S9 File Protein RMSF of 8H64. (XLSX)

S10 File Post-simulation MM-GBSA (4CDB). (XLSX)

S11 File Post-simulation MM-GBSA (8H64). (XLSX)
