# Supplementary material for: Integrative computational analysis of plant-derived flavonoids as inhibitors of Listeriolysin O and Internalin A in Listeria monocytogenes
Source: PLoS One. 2026 Jun 9;21(6):e0351129. doi: 10.1371/journal.pone.0351129 (PMC13249230; doi:10.1371/journal.pone.0351129)
Supplement: S1 File — (DOCX) [file pone.0351129.s001.docx]

**Table 1.** 4CDB protein; physicochemical characteristics, lipophilicity, water solubility, gastrointestinal absorption, and accessibility of specific synthesis of three lead compounds (CIDs: 441667, 15126294, 187808) with control compound (CID 6249).

| Properties | | CID 441667 | CID 15126294 | CID 187808 | CID 6249 |
| --- | --- | --- | --- | --- | --- |
| Physico-chemical Properties | MW (g/mol) | 449.4 | 449.4 | 446.4 | 349.4 |
|  | Rotatable bonds | 4 | 4 | 5 | 4 |
|  | H-bond acceptors | 10 | 10 | 10 | 5 |
|  | H-bond donors | 8 | 8 | 5 | 3 |
| Lipophilicity | Consensus Log Po/w | 0.382 | 0.382 | 0.3529 | 0.3181 |
| Water Solubility | Log S (ESOL) | -2.929 | -2.942 | -2.902 | -2.396 |
| Pharmacokinetics | GI absorption | 45.392 | 38.6 | 51.094 | 43.034 |
| Drug likeness | Lipinski, Violation | 1 | 1 | 0 | 0 |

**Table 2.** 8H64 protein; physicochemical characteristics, lipophilicity, water solubility, gastrointestinal absorption, and accessibility of specific synthesis of three lead compounds (CIDs: 441699, 443648, 442868) with control compound (CID 6249).

| Properties | | CID 441699 | CID 443648 | CID 442868 | CID 6249 |
| --- | --- | --- | --- | --- | --- |
| Physico-chemical Properties | MW (g/mol) | 449.4 | 433.4 | 383.4 | 349.4 |
|  | Rotatable bonds | 4 | 4 | 3 | 4 |
|  | H-bond acceptors | 10 | 9 | 7 | 5 |
|  | H-bond donors | 8 | 7 | 3 | 3 |
| Lipophilicity | Consensus Log Po/w | 0.382 | 0.6764 | 3.3521 | 0.3181 |
| Water Solubility | Log S (ESOL) | -2.929 | -2.816 | -3.557 | -2.396 |
| Pharmacokinetics | GI absorption | 45.392 | 48.354 | 84.549 | 43.034 |
| Drug likeness | Lipinski, Violation | 1 | 1 | 0 | 0 |
