# Supplementary material for: Integrative computational analysis of plant-derived flavonoids as inhibitors of Listeriolysin O and Internalin A in Listeria monocytogenes
Source: PLoS One. 2026 Jun 9;21(6):e0351129. doi: 10.1371/journal.pone.0351129 (PMC13249230; doi:10.1371/journal.pone.0351129)
Supplement: S2 File — (DOCX) [file pone.0351129.s002.docx]

**Table 1.** 4CDB protein; Comparison of the predicted toxicity profiles of three selected compounds (CID 441667, CID 15126294, and CID 187808) with those of a control ligand (CID 6249).

| **Classification** | **Target** |  | **CID: 441667** | **CID: 15126294** | **CID: 187808** | **CID: 6249 (control)** |
| --- | --- | --- | --- | --- | --- | --- |
| **Anticipated LD50** | |  | 5000mg/kg | 5000mg/kg | 2500mg/kg | 5000mg/kg |
| **Predicted toxicity class** | |  | 5 | 5 | 5 | 5 |
| **Organ Toxicity** | Hepatotoxicity | Prediction | Inactive | Inactive | Inactive | Inactive |
|  |  | Probability | (0.82) | (0.82) | (0.83) | (0.87) |
|  | Neurotoxicity | Prediction | Inactive | Inactive | Inactive | Inactive |
|  |  | Probability | (0.89) | (0.89) | (0.86) | (0.87) |
|  | Nephrotoxicity | Prediction | Active | Active | Active | Inactive |
|  |  | Probability | (0.73) | (0.73) | (0.76) | (0.84) |
|  | Respiratory toxicity | Prediction | Active | Active | Active | Active |
|  |  | Probability | (0.59) | (0.59) | (0.61) | (0.96) |
|  | Cardiotoxicity | Prediction | Inactive | Inactive | Inactive | Inactive |
|  |  | Probability | (0.63) | (0.63) | (0.63) | (0.82) |
| **Toxicity end points** | Carcinogenicity | Prediction | Inactive | Inactive | Inactive | Inactive |
|  |  | Probability | (0.84) | (0.84) | (0.90) | (0.83) |
|  | Immunotoxicity | Prediction | Active | Inactive | Active | Inactive |
|  |  | Probability | (0.64) | (0.52) | (0.87) | (0.98) |
|  | Mutagenicity | Prediction | Inactive | Inactive | Inactive | Inactive |
|  |  | Probability | (0.72) | (0.72) | (0.65) | (0.94) |
|  | Cytotoxicity | Prediction | Inactive | Inactive | Inactive | Inactive |
|  |  | Probability | (0.61) | (0.61) | (0.58) | (0.60) |
|  | BBB-barrier | Prediction | Active | Active | Inactive | Inactive |
|  |  | Probability | (0.53) | (0.53) | (0.80) | (1.0) |
|  | Ecotoxicity | Prediction | Inactive | Inactive | Inactive | Inactive |
|  |  | Probability | (0.59) | (0.59) | (0.61) | (0.75) |
|  | Clinical toxicity | Prediction | Inactive | Inactive | Active | Active |
|  |  | Probability | (0.69) | (0.69) | (0.53) | (0.53) |
|  | Nutritional toxicity | Prediction | Active | Active | Active | Active |
|  |  | Probability | (0.53) | (0.53) | (0.51) | (0.50) |

**Table 2.** 8H64 protein; Comparison of the predicted toxicity profiles of three selected compounds (CID 441699, CID 443648, and CID 442868) with those of a control ligand (CID 6249).

| **Classification** | **Target** |  | **CID: 441699** | **CID: 443648** | **CID: 442868** | **CID: 6249 (control)** |
| --- | --- | --- | --- | --- | --- | --- |
| **Anticipated LD50** | |  | 5000mg/kg | 5000mg/kg | 161 mg/kg | 5000mg/kg |
| **Predicted toxicity class** | |  | 5 | 5 | 5 | 5 |
| **Organ Toxicity** | Hepatotoxicity | Prediction | Inactive | Inactive | Inactive | Inactive |
|  |  | Probability | (0.82) | (0.82) | (0.81) | (0.87) |
|  | Neurotoxicity | Prediction | Inactive | Inactive | Active | Inactive |
|  |  | Probability | (0.89) | (0.89) | (0.56) | (0.87) |
|  | Nephrotoxicity | Prediction | Active | Active | Inactive | Inactive |
|  |  | Probability | (0.73) | (0.73) | (0.54) | (0.84) |
|  | Respiratory toxicity | Prediction | Active | Active | Active | Active |
|  |  | Probability | (0.59) | (0.59) | (0.93) | (0.96) |
|  | Cardiotoxicity | Prediction | Inactive | Inactive | Inactive | Inactive |
|  |  | Probability | (0.63) | (0.63) | (0.69) | (0.82) |
| **Toxicity end points** | Carcinogenicity | Prediction | Inactive | Inactive | Inactive | Inactive |
|  |  | Probability | (0.84) | (0.84) | (0.59) | (0.83) |
|  | Immunotoxicity | Prediction | Active | Inactive | Active | Inactive |
|  |  | Probability | (0.64) | (0.65) | (0.98) | (0.98) |
|  | Mutagenicity | Prediction | Inactive | Inactive | Inactive | Inactive |
|  |  | Probability | (0.72) | (0.72) | (0.55) | (0.94) |
|  | Cytotoxicity | Prediction | Inactive | Inactive | Inactive | Inactive |
|  |  | Probability | (0.61) | (0.61) | (0.61) | (0.60) |
|  | BBB-barrier | Prediction | Active | Active | Active | Inactive |
|  |  | Probability | (0.53) | (0.53) | (0.73) | (1.0) |
|  | Ecotoxicity | Prediction | Inactive | Inactive | Inactive | Inactive |
|  |  | Probability | (0.59) | (0.59) | (0.55) | (0.75) |
|  | Clinical toxicity | Prediction | Inactive | Inactive | Active | Active |
|  |  | Probability | (0.69) | (0.69) | (0.61) | (0.53) |
|  | Nutritional toxicity | Prediction | Active | Active | Active | Active |
|  |  | Probability | (0.53) | (0.53) | (0.53) | (0.50) |
