# Supplementary material for: Integrative computational analysis of plant-derived flavonoids as inhibitors of Listeriolysin O and Internalin A in Listeria monocytogenes
Source: PLoS One. 2026 Jun 9;21(6):e0351129. doi: 10.1371/journal.pone.0351129 (PMC13249230; doi:10.1371/journal.pone.0351129)
Supplement: S3 File — (DOCX) [file pone.0351129.s003.docx]

**Table 1.** The amino acid-binding residues between the 4CDB and three lead compounds along with a control compound found during the XP molecular docking.

| **PubChem CID** | **Compound Name** | **H-Bond** | **Polar Bond** | **Hydrophobic Bond** |
| --- | --- | --- | --- | --- |
| 441667 | Cyanidin 3-glucoside | GLU 209,  GLU 408,  GLN 245,  TYR 406 | GLN 216,  GLN 245,  THR 313,  SER 312,  THR 410 | TYR 212,  TYR 406,  MET 210,  VAL 315 |
| 15126294 | Cyanidin 5-O-glucoside | GLU 209,  SER 107,  TYR 212,  GLN 245,  LYS 106 | ASN 109,  SER 107,  SER 312,  GLN 245,  THR 313,  THR 410 | ILE 108,  MET 210,  TYR 212,  VAL 315,  TYR 406 |
| 187808 | Glycitein 7-O-glucoside | GLU 209,  TYR 406,  SER 411,  LYS 106 | GLN 245,  GLN 216,  THR 313,  THR 410,  SER 312,  SER 411,  HIS 311 | MET 210,  TYR 212,  TYR 406 |
| 6249 (Control) | Ampicillin | GLU 209 | GLN 110,  ASN 109,  SER 107,  GLN 216 | ILE 108,  MET 210,  TYR 212,  TYR 406 |

**Table 2.** The amino acid-binding residues between the 8H64 and three lead compounds along with a control compound found during the XP molecular docking.

| **PubChem CID** | **Compound Name** | **H-Bond** | **Polar Bond** | **Hydrophobic Bond** |
| --- | --- | --- | --- | --- |
| 441699 | Cyanidin 3-O-galactoside | ASP 279.  LYS 301,  GLU 326,  TYR 347,  ARG 365 | SER 257,  ASN 259,  ASN 325,  THR 237,  THR 345 | ALA 281,  ALA 304,  PHE 367,  TYR 343,  TYR 347 |
| 443648 | Pelargonidin 3-glucoside ion | LYS 301,  ASP 279,  GLU 326,  ARG 365 | ASN 259,  SER 257,  THR 237,  ASN 325,  THR 345 | ALA 281,  ALA 304,  TYR 347,  TYR 343,  PHE 367 |
| 442868 | Phyllospadine | SER 216,  ARG 211,  GLU 255 | SER 172,  SER 215,  SER 216,  ASN 238,  THR 237,  SER 257,  ASN 259 | ILE 235 |
| 6249 (Control) | Ampicillin | THR 237,  LYS 301,  ASP 277,  GLU 255,  SER 257 | THR 237,  ASN 259,  SER 257 | ILE 235,  LEU 256 |
